# Probing the electronic and mechanistic roles of the μ_4_-sulfur atom in a synthetic Cu_Z_ model system†

**DOI:** 10.1039/c9sc06251c

**Published:** 2020-02-17

**Authors:** Suresh C. Rathnayaka, Shahidul M. Islam, Ida M. DiMucci, Samantha N. MacMillan, Kyle M. Lancaster, Neal P. Mankad

**Affiliations:** Department of Chemistry, University of Illinois at Chicago 845 W. Taylor St. Chicago IL 60607 USA npm@uic.edu; Department of Chemistry & Chemical Biology, Cornell University, Baker Laboratory Ithaca NY 14853 USA kml236@cornell.edu

## Abstract

Nitrous oxide (N_2_O) contributes significantly to ozone layer depletion and is a potent greenhouse agent, motivating interest in the chemical details of biological N_2_O fixation by nitrous oxide reductase (N_2_OR) during bacterial denitrification. In this study, we report a combined experimental/computational study of a synthetic [4Cu:1S] cluster supported by N-donor ligands that can be considered the closest structural and functional mimic of the Cu_Z_ catalytic site in N_2_OR reported to date. Quantitative N_2_ measurements during synthetic N_2_O reduction were used to determine reaction stoichiometry, which in turn was used as the basis for density functional theory (DFT) modeling of hypothetical reaction intermediates. The mechanism for N_2_O reduction emerging from this computational modeling involves cooperative activation of N_2_O across a Cu/S cluster edge. Direct interaction of the μ_4_-S ligand with the N_2_O substrate during coordination and N–O bond cleavage represents an unconventional mechanistic paradigm to be considered for the chemistry of Cu_Z_ and related metal–sulfur clusters. Consistent with hypothetical participation of the μ_4_-S unit in two-electron reduction of N_2_O, Cu K-edge and S K-edge X-ray absorption spectroscopy (XAS) reveal a high degree of participation by the μ_4_-S in redox changes, with approximately 21% S 3p contribution to the redox-active molecular orbital in the highly covalent [4Cu:1S] core, compared to approximately 14% Cu 3d contribution per copper. The XAS data included in this study represent the first spectroscopic interrogation of multiple redox levels of a [4Cu:1S] cluster and show high fidelity to the biological Cu_Z_ site.

## Abstract 2

Experimental data and computational modeling indicates an active role for the bridging sulfide ligand in a synthetic Cu_Z_ model.

## Graphical abstract

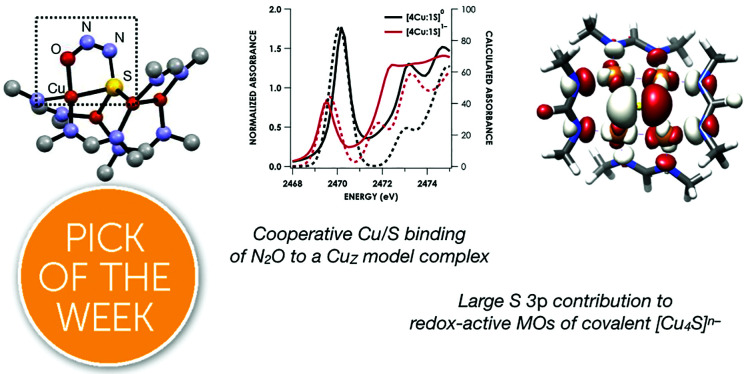

## Introduction

Metal–sulfide clusters represent a common motif in bioinorganic chemistry. The most studied examples are iron–sulfur clusters (*e.g.* [2Fe:2S], [4Fe:4S], *etc.*) that serve as ubiquitous electron transfer sites in a wide range of metalloproteins.^1^ Other scenarios such as the [NiFe] and [MoCu] catalytic sites of carbon monoxide dehydrogenases (CODHs),^2,3^ the H-cluster found in [FeFe] hydrogenases, and the [FeMo]-cofactor of nitrogenases^4^ involve multinuclear metal–sulfide clusters facilitating multielectron/multiproton catalytic transformations. Typically, the bridging sulfido (S^2−^) ligands in these clusters are thought to be crucial for electronically coupling the transition metal sites, thereby facilitating electron delocalization and lowering barriers towards electron transfer either to/from a catalytic site or along an electron transport chain. However, only in rare cases are the sulfur centers proposed to play a direct rather than spectator role with regard to bond activation and/or bond formation. In the case of the [FeMo]-cofactor of nitrogenase, various hypotheses have been put forward in which sulfide ligands might act as redox-active proton relays^5^ or even that sulfide(s) may serve in a gating mechanism to nitrogenase activity.^6^ In the case of the [MoCu] catalytic site of aerobic CODH, one mechanistic hypothesis based on crystallographic studies with substrate analogues involves the μ_2_-sulfide ligand actively participating in CO activation *via* transient S–C bond formation.^2^

**Fig. 1:**
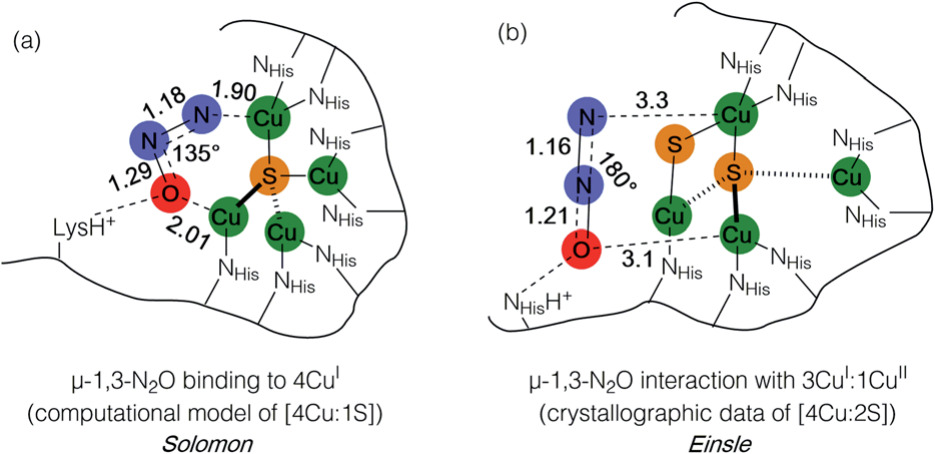
N_2_O interactions with the Cu_Z_ site of nitrous oxide reductase proposed by (a) Solomon and (b) Einsle. Distances are given in Å.

During bacterial denitrification, nitrous oxide (N_2_O) is converted to N_2_ + H_2_O in a 2e^−^/2H^+^ reaction catalyzed by the metalloenzyme, nitrous oxide reductase (N_2_OR).^7^ The catalytic site of N_2_OR is a tetranuclear copper–sulfur cluster, Cu_Z_, which has been structurally characterized in both [4Cu:1S] and [4Cu:2S] forms.^8,9^ Both forms show N_2_O reductase activity to some extent, and both require physiological reduction to their most reduced redox states to activate N_2_O: the 4Cu^I^ (“fully reduced”) state for the [4Cu:1S] cluster and the 3Cu^I^:1Cu^II^ (“1-hole”) state for the [4Cu:2S] cluster.^10^ For the [4Cu:1S] cluster, Solomon has proposed N_2_O binding across a dicopper(i) cluster edge, with the N_2_O molecule occupying a μ-1,3 binding mode, based on computational modeling (Fig. 1a).^11^ For the [4Cu:2S] form, Einsle has reported crystallographic data on N_2_O-pressurized crystals of N_2_OR showing a N_2_O molecule within van der Waals contact of Cu_Z_, but the N_2_O molecule was not found within coordination distance of Cu_Z_ and had not undergone significant activation (Fig. 1b).^9^ In neither case has experimental data emerged to probe the nature of N_2_O activation by the copper–sulfur clusters.

**Scheme 1:**
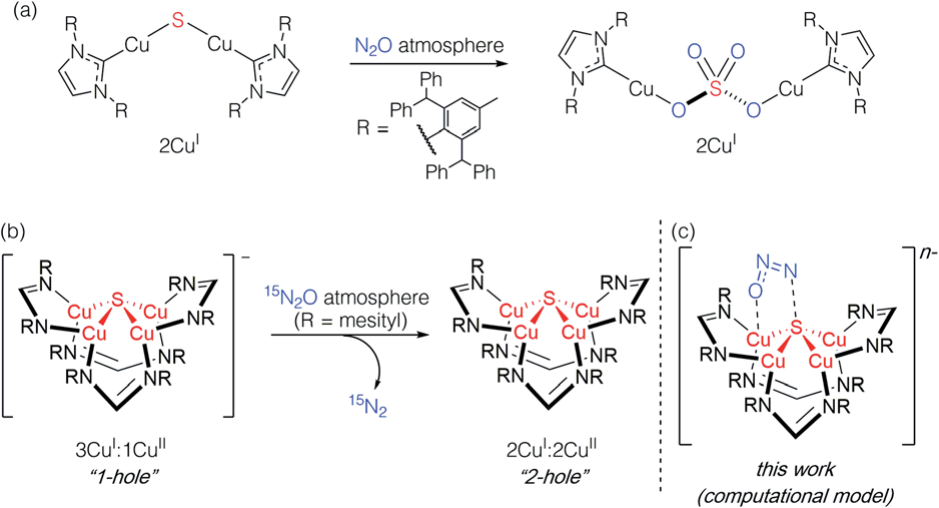
Active participation of bridging sulfide ligands in N_2_O activation by (a) binuclear and (b) and (c) tetranuclear synthetic copper sulfide models.

Studying synthetic model systems can aid understanding of how these unusual inorganic copper–sulfur functional groups behave,^12^ which is particularly crucial knowledge in the context of N_2_O's significant impact as a greenhouse gas and an ozone layer depleting agent.^13,14^ Among the synthetic copper compounds and materials known to activate N_2_O,^15–18^ one of our groups has reported the only examples of N_2_O activation by copper sulfide clusters. In one case, a dicuprous [Cu_2_S] cluster with an unsupported μ_2_-sulfide bridge^19^ was found to reduce multiple N_2_O equivalents to N_2_, resulting in exhaustive oxidation of the sulfur center to a μ_2_-sulfate ligand (Scheme 1a).^20^ Here, the copper centers remained redox inactive while the μ_2_-sulfide ligand was not only the redox-active center but also acted as an oxygen atom acceptor. In another case, a phosphine-supported tetranuclear [Cu_4_S] cluster in its 4Cu^I^ state showed reactivity towards N_2_O reduction,^21^ but the cluster lost structural integrity during the reaction, losing the sulfur atom to unknown products in the reaction medium and thus limiting insight that can be gained about its role. Finally, a formamidinate-supported [Cu_4_S] cluster in its formally 3Cu^I^:1Cu^II^ ([4Cu:1S]^1−^) state was found to reduce ^15^N_2_O to ^15^N_2_ (Scheme 1b).^22,23^ Here the μ_4_-sulfide bridge remained intact during a formal oxidation to the 2Cu^I^:2Cu^II^ ([4Cu:1S]^0^) redox state of the cluster, allowing us to establish a closed cycle for N_2_O reduction. Based on these results, the potential role (or lack thereof) of the bridging sulfide ligand in copper–sulfur clusters merits further investigation.

In this report, we disclose a combined experimental/computational study of the latter system that collectively implicates the μ_4_-sulfide ligand as participating in redox changes and directly interacting with N_2_O during its activation (Scheme 1c). Our data includes the first spectroscopic interrogation of multiple [4Cu:1S] redox levels, which has proven challenging in the metalloenzyme system,^7,24^ and highlights the fidelity of our synthetic model to the biological Cu_Z_ site. Additionally, the direct interaction of N_2_O with the bridging sulfur atom(s) in Cu_Z_ has not been proposed before. Such reaction pathways should be considered for the chemistry of Cu_Z_ and related metal–sulfide clusters in light of the synthetic model studies reported herein.

## Results and discussion

**Scheme 2:**
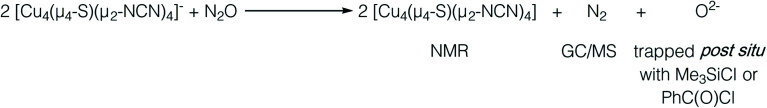
Overall balanced reaction being studied.

In our previous report of N_2_O reduction by the anionic complex [Cu_4_(μ_4_-S)(μ_2_-NCN)_4_]^1−^ (the 1-hole cluster, referred to here as [4Cu:1S]^1−^) as its [K(18-crown-6)]^+^ salt (NCN = [MesNC(H)NMes]^1−^),^22^ we were able to use NMR spectroscopy, isotopic labeling experiments, and *post situ* electrophilic trapping to establish the presence of three products: neutral [Cu_4_(μ_4_-S)(μ_2_-NCN)_4_] (the 2-hole cluster, referred to here as [4Cu:1S]^0^), N_2_, and O^2−^. However, we were unable to definitively establish the reaction stoichiometry at that time. Since then, we have undertaken quantitative GC-MS analysis of the reaction headspace to determine the yield of N_2_. According to this analysis (see ESI†), 0.53 ± 0.06 mol of N_2_ are produced per mol of the [4Cu:1S]^1−^ complex. When combining this result with our previous observations, we can now confidently propose the balanced reaction shown in Scheme 2 as the dominant pathway. Based on this reaction stoichiometry, we proceeded with the working hypothesis that one equivalent of [4Cu:1S]^1−^ is responsible for N_2_O activation while a second equivalent is acting as a sacrificial reductant, thus accounting for the overall two-electron redox reaction.

**Fig. 2:**
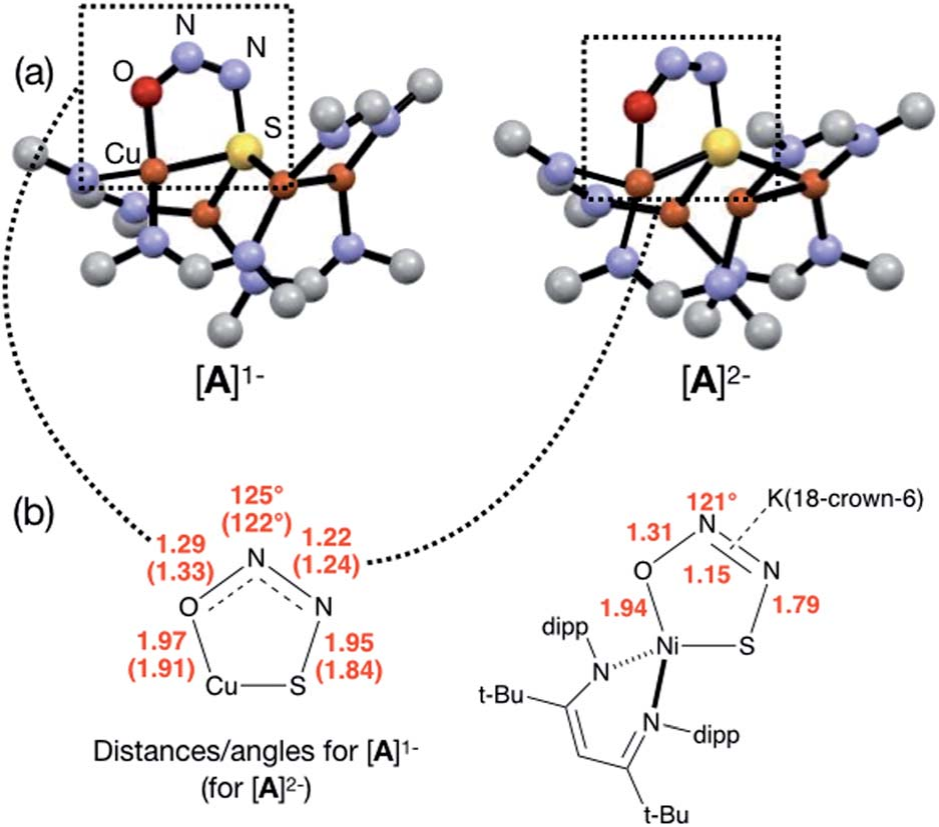
(a) Optimized structures of N_2_O-activated intermediates [**A**]^1−^ and [**A**]^2−^ (color code: Cu, orange; S, yellow; O, red; N, blue; C, gray). (b) Comparison of the cyclic core structures of [**A**]^*n*−^ and a related mononuclear Ni complex characterized by Hayton;^25^ distances are given in Å.

Next, because we have been unable to detect any intermediates experimentally, we sought to examine the binding mode of N_2_O using DFT modeling at the B3LYP/6-31G(d) level in the gas phase. To save computational time, the mesityl groups on the supporting NCN ligands were replaced with methyl groups. After attempting to simulate several types of adducts between the [4Cu:1S]^1−^ model complex and N_2_O, we were able to locate minima associated with N_2_O coordination to both the [4Cu:1S]^1−^ model (intermediate [**A**]^1−^) and to its closed-shell, fully reduced [4Cu:1S]^2−^ analogue (intermediate [**A**]^2−^). In both cases N_2_O occupied a μ-1,3 binding mode, but to our surprise the N_2_O molecule was found to bridge one of the Cu centers and the S atom (Fig. 2a). In each case, one of the other Cu centers has moved away from the S atom to facilitate its direct interaction with N_2_O. An alternative, μ_3_-1,2 binding mode in which the N_2_O molecule bridges two Cu centers as well as the S atom also was located but was determined to be significantly higher in energy by +11.8 kcal mol^−1^ on the Gibbs free energy surface (see ESI†). The preferred binding mode for this model system is distinct from the μ-1,3 bridging between two Cu centers that is proposed for Cu_Z_ (see Fig. 1a), where the μ_4_-S^2−^ ligand is not proposed to interact directly with N_2_O. It should be noted that a mononuclear intermediate in which N_2_O bridges across a terminal nickel–sulfide bond has been isolated and crystallographically characterized by Hayton and coworkers.^25,26^ The accord between the metrical parameters of the activated N_2_O in our computational model with Hayton's experimental data (Fig. 2b) lends further support to the intermediacy of [**A**]^1−^. Binding of N_2_O to the 1-hole [4Cu:1S]^1−^ model to form [**A**]^1−^ was calculated to be endothermic by +18.5 kcal mol^−1^, consistent with our inability to observe an N_2_O-bound intermediate experimentally.

**Scheme 3:**
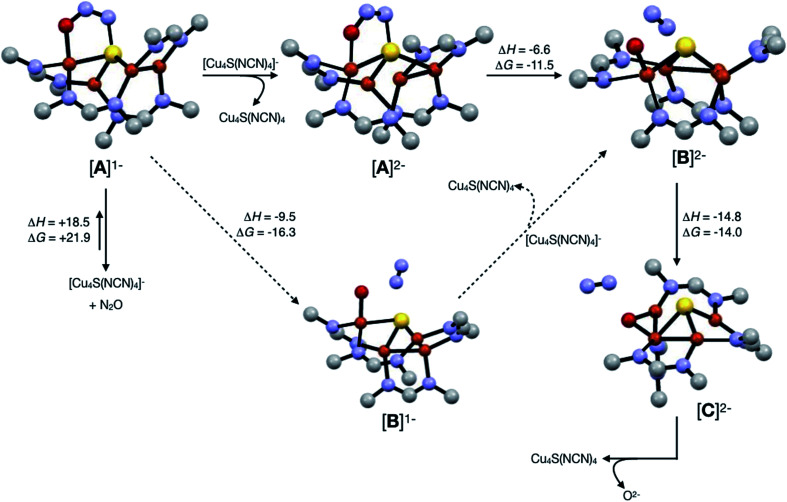
Reaction pathways modeled by DFT (B3LYP/6-31G(d)). Energies at 298 K are shown in kcal mol^−1^. The favored pathway is shown with solid arrows, and the disfavored pathway with dotted arrows.

Assuming that small equilibrium concentrations of an N_2_O-bound intermediate akin to [**A**]^1−^ form under N_2_O atmosphere, we next considered the potential reaction pathway to N_2_ + O^2−^ (Scheme 3). We expect that N_2_O binding to the [4Cu:1S]^1−^ complex would raise its reduction potential, due to the π-accepting nature of N_2_O.^27^ Thus, there would be a driving force for [**A**]^1−^ to undergo reduction by a sacrificial 1-hole complex to provide [**A**]^2−^. As in the [4Cu:1S] form of Cu_Z_,^11^ [**A**]^2−^ is in the fully-reduced 4Cu^I^ state and thus is expected to π-backdonate sufficient electron density into the N_2_O π* manifold to induce N–O bond cleavage. Conversion to the resulting intermediate [**B**]^2−^ from N_2_ loss was calculated to be exothermic relative to [**A**]^2−^. Further energy lowering was found by shifting the terminal O^2−^ ligand in [**B**]^2−^ to a μ_2_-bridging position in [**C**]^2−^. In the case of Cu_Z_, Solomon has reported that the on-cycle intermediate Cu_Z_° formed after N_2_ loss features a terminal oxygen ligand stabilized by hydrogen bonding with a nearby lysine residue, and has found that disruption of hydrogen bonding produces the off-cycle intermediate Cu_Z_* in which the oxygen ligand occupies its thermodynamically preferred bridging position.^11^ Because we propose O^2−^ to be a stoichiometric product of our aprotic model reaction, we assume that O^2−^ dissociates from either [**B**]^2−^ or [**C**]^2−^.

An alternative pathway (Scheme 3, dotted arrows) would involve N_2_ loss directly from 1-hole [**A**]^1−^ prior to reduction, producing intermediate [**B**]^1−^. Reduction of [**B**]^1−^ by a sacrificial [4Cu:1S]^1−^ complex would then produce intermediate [**B**]^2−^ that is common to both pathways. However, because O^2−^ is expected to lower the reduction potential of the tetracopper cluster due to its π-donor character, it should be unfavorable for [**B**]^1−^ to undergo reduction by the sacrificial 1-hole species. Indeed, [**A**]^1−^ was calculated to be more oxidizing than [**B**]^1−^ by 0.21 V. Thus, we consider this alternative pathway to be unlikely, but we cannot rule it out definitively.

Because the μ_4_-sulfide ligand seems to play a crucial and direct role in N_2_O activation according to our DFT modeling, we wondered whether the frontier orbitals of these synthetic [4Cu:1S] complexes have notable sulfur character. In order to validate our mechanistic model, we thus undertook multi-edge X-ray absorption spectroscopy (XAS) combined with higher-level computational modeling to interrogate the electronic structural changes underpinning the [4Cu:1S]^0/1−^ redox process.

**Fig. 3:**
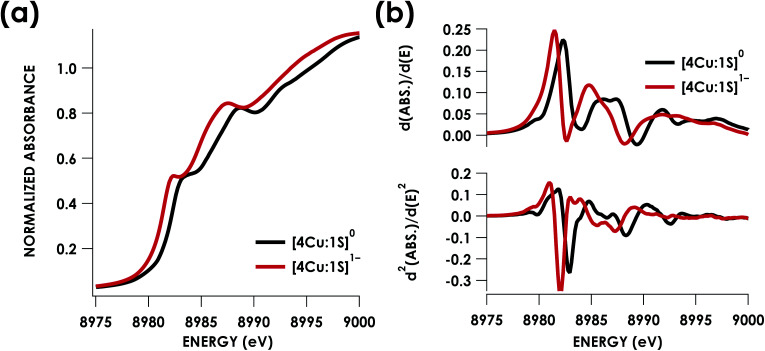
(a) Cu K-edge XAS spectra obtained for the [4Cu:1S]^0/1−^ redox couple. (b) First (top) and second (bottom) derivative Cu K-edge XAS spectra. Isoenergetic pre-edge (1s → 3d) excitations are evident in the second-derivative spectra at 8979.8 eV. Rising edge inflection points occur at 8982.3 and 8985.9 eV for [4Cu:1S]^0^ and 8981.5 and 8984.8 eV for [4Cu:1S]^1−^.

Cu K-edge XAS data obtained for [4Cu:1S]^1−^ and [4Cu:1S]^0^ are shown in Fig. 3a. Spectral subtraction was carried out to remove a minor contribution of [4Cu:1S]^0^ in the spectrum of the monoanion (*vide infra*). Neither spectrum presents a resolved pre-edge (1s → 3d) feature, although both spectra feature a shoulder that gives a peak in the second derivative spectrum at 8979.8 eV, consistent with the presence of Cu 3d vacancies (Fig. 3b). The rising edges of the two spectra have qualitatively similar fine structure including maxima at *ca.* 8983 eV suggesting the presence of Cu^I^ centers,^28^ although the spectrum of the [4Cu:1S]^1−^ cluster is shifted, with inflection points occurring at 0.8 to 1.1 eV lower energy relative to [4Cu:1S]^0^. Given the effectively identical coordination environments between the two species, the shift in rising edge position largely reflects some Cu participation in the redox process. Moreover, the lack of dramatic intensity changes for the rising edge features suggests a delocalized redox process, *i.e.* that a localized [2Cu^I^:2Cu^II^]/[3Cu^I^:1Cu^II^] description is not appropriate.

**Fig. 4:**
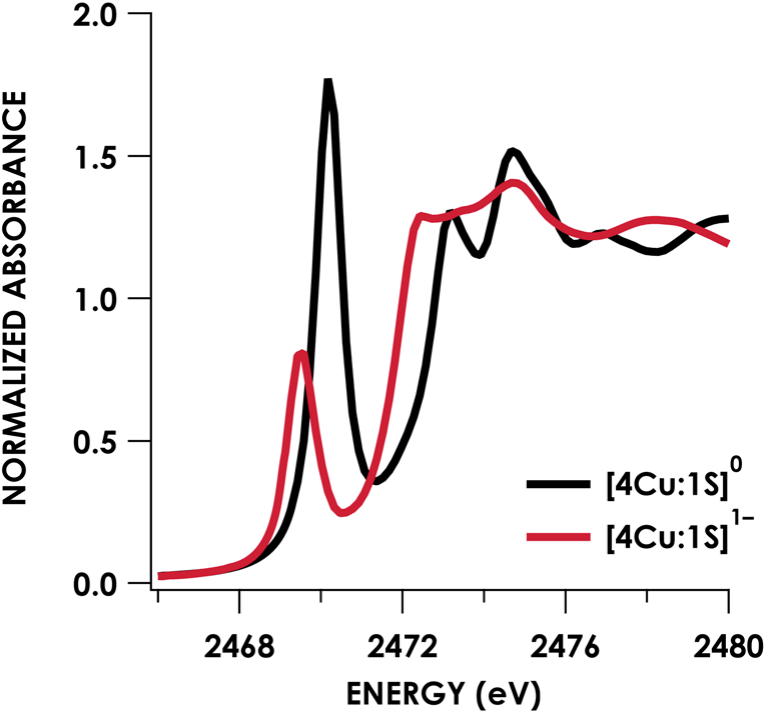
S K-edge XAS data obtained for the [4Cu:1S]^0/1−^ redox couple. Pre-edge peaks corresponding to S 1s → ψ* excitations are located at 2470.2 eV for [4Cu:1S]^0^ and 2469.5 eV for [4Cu:1S]^1−^.

Quantitative estimates of S participation in the redox-active molecular orbital (RAMO) can be gleaned through analysis of S K-edge XAS data^29^ obtained for the two clusters, which are presented in Fig. 4. Well-resolved pre-edge peaks are apparent in both spectra, occurring at 2470.2 eV for [4Cu:1S]^0^ and 2469.5 eV in the spectrum of [4Cu:1S]^1−^. A *ca.* 18% [4Cu:1S]^0^ impurity was evident in the spectrum of [4Cu:1S]^1−^ which was removed by subtraction and re-normalization as carried out by Solomon and co-workers to remove S K-edge XAS contributions from Cu_A_ in N_2_OR^30,31^ (Fig. S15†). Notably, the 2469.5 eV [4Cu:1S]^1−^ pre-edge peak energy value closely matches pre-edge peak energies reported by Solomon and co-workers for the Cu_Z_ sites of resting *Achromobacter cycloclastes*^31^ and *Paracoccus denitrificans*^30^ N_2_OR at 2469.2 and 2469.0 eV, respectively. On the basis of Cu K-edge XAS analysis, Solomon and co-workers assigned resting Cu_Z_ as a 3Cu^I^:1Cu^II^ cluster,^30^ consistent with the formal oxidation state distribution expected for the [4Cu:1S]^1−^ cluster.

Pre-edge peaks in the S K-edge XAS spectra of metal complexes and clusters bearing S-donor ligands reflect excitations from S 1s → ψ*, where ψ*, the acceptor MO, is an anti-bonding ligand field MO born of metal–sulfur mixing:

1 ψ* ≈ *α*^2^S 3p − (1 − *α*)^2^M 3d

where *α*^2^ reflects the % 3p contribution in the acceptor MO.^29^

Pre-edge peak intensities (*D*_0_) are then given by the relationship:2
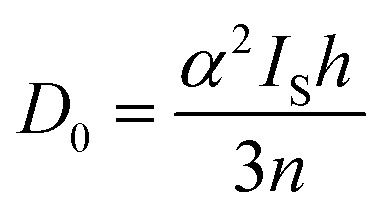
where *h* is the number of holes in the acceptor MO, *n* is the number of photoabsorbing nuclei from which electrons can be excited into the acceptor MO, and *I*_s_ is the radial dipole integral 〈3p|r|1s〉 governing the intensity of a “pure” S 1s → 3p excitation. Solomon and co-workers^32^ have estimated the value of *I*_s_ as a function of the S 1s → 4p excitation energy, which can itself be gleaned from S K-edge XAS data and will vary according to the nature of the S photoabsorber and its chemical environment. Using TDDFT calculations to facilitate the assignments (*vide infra*), the S 1s → 4p transition for [4Cu:1S]^0^ occurs at 2477.0 eV and at 2475.9 eV for [4Cu:1S]^1−^. Using the relationship from Solomon and co-workers,^32^ the value of *I*_s_ for [4Cu:1S]^0^ is 14.9 and is 12.9 for [4Cu:1S]^1−^.

Fitting pseudo-Voigt peaks to the pre-edge peaks in the S K-edge data give integrated peak areas *D*_0_ for the two clusters of 2.03 ± 0.01 for [4Cu:1S]^0^ and 0.91 ± 0.02 for [4Cu:1S]^1−^. The *ca.* twofold decrease in *D*_0_ upon reduction confirms S 3p contribution to the RAMO shared by the redox couple. Application of eqn (2) then gives 20.5 ± 0.1% S 3p in the RAMO of [4Cu:1S]^0^ and 21.1 ± 0.5% S 3p in the RAMO of [4Cu:1S]^1−^. The latter values are comparable to the estimate given by Solomon and co-workers for the RAMO of the Cu_Z_ site in resting *Paracoccus denitrificans* N_2_OR at 15–22%.^30^

**Fig. 5:**
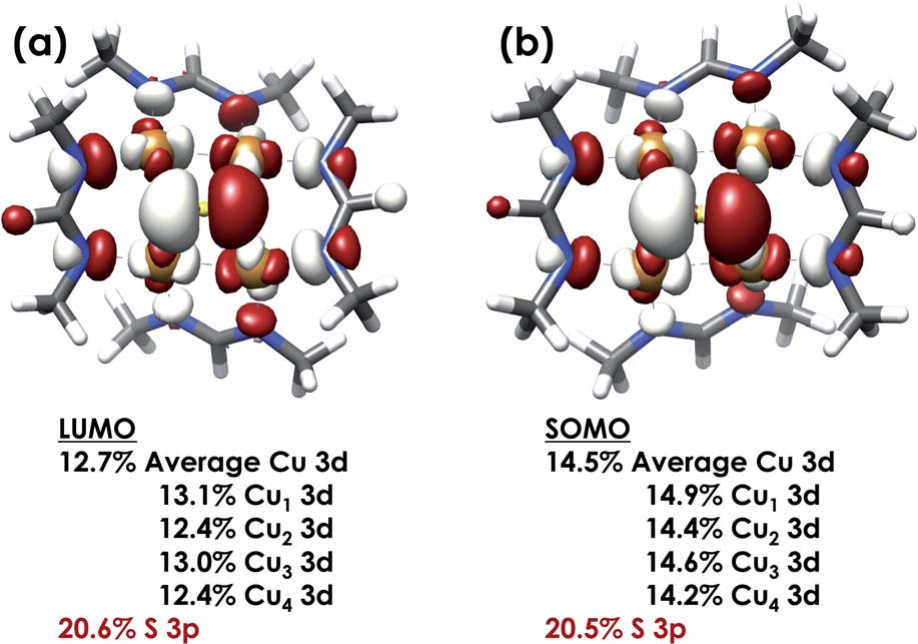
(a) Restricted Kohn–Sham LUMO for [4Cu:1S]^0^ and (b) quasi-restricted (QRO)^33^ SOMO for [4Cu:1S]^1−^. Both MOs were calculated for truncated models using the B3LYP hybrid density functional with the CP(PPP) basis set on Cu and the scalar-relativistically recontracted ZORA-def2-TZVP(-f) basis set on all other atoms. Orbitals are plotted at an isovalue of 0.03 au.

**Fig. 6:**
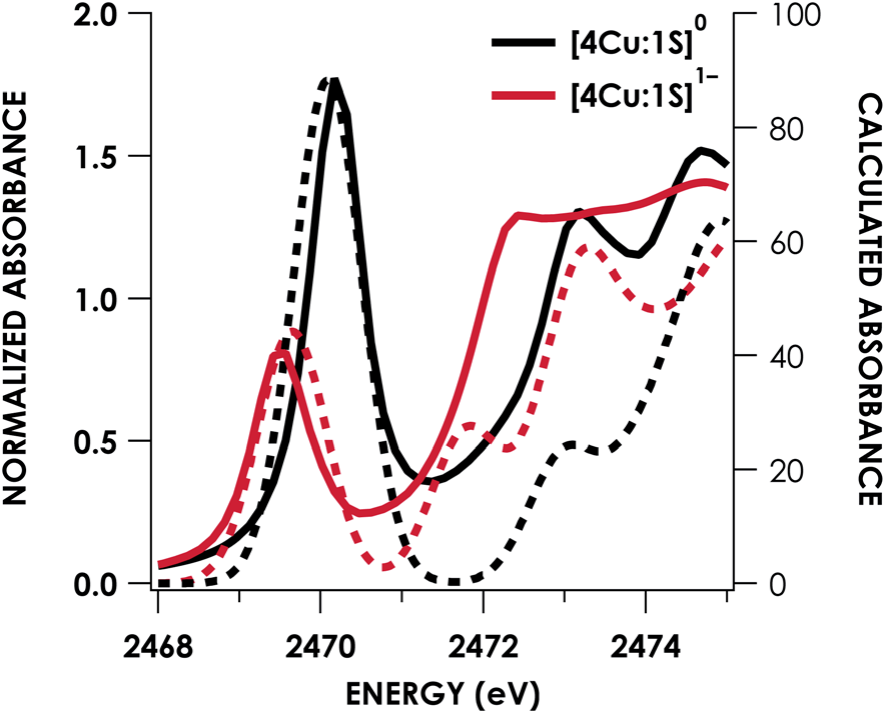
Overlay of TDDFT-calculated (dashed) S K-edge XAS with experimental (solid) spectra obtained for the [4Cu:1S]^0/1−^ redox series. TDDFT calculations were initiated from B3LYP single-point calculations with the CP(PPP)^34^ basis set on Cu and the ZORA-def2-TZVP(-f)^38^ basis set on all other atoms. Calculated spectra are shifted by +40.4 eV to correct for inaccurate core potential modeling endemic to standard hybrid DFT calculations.

DFT calculations were carried out to further interrogate the nature of the RAMO in the [4Cu:1S]^0/1−^ redox couple. Calculations were carried out on truncated models as described above and employed the B3LYP hybrid density functional with the CP(PPP) basis set^34,35^ on Cu and the scalar relativistically recontracted ZORA-def2-TZVP(-f)^36^ basis on all other atoms. The LUMO of [4Cu:1S]^0^ and SOMO of [4Cu:1S]^1−^ are depicted in Fig. 5. These are qualitatively similar, indicating that the RAMO is a highly delocalized orbital featuring effectively equal participation of Cu 3d from all 4 metal centers along with a significant contribution from S 3p. Equal participation of all three Cu centers in the SOMO was previously indicated by simulation of experimental EPR parameters.^22^ The equal contributions from Cu are also in accord with observation that the Cu K-edge XANES shift in energy but do not exhibit differences in fine structure. Calculated S 3p contributions are 20.6% for [4Cu:1S]^0^ and 21.1% for [4Cu:1S]^1−^, in splendid agreement with experiment as well as with previous EPR analysis of the [4Cu:1S]^1−^ species that indicated anomalously small Cu hyperfine coupling.^22^ Moreover, TDDFT calculations^37^ of the S K-edge XAS for both species initiated from the aforementioned single-point DFT calculations give spectra that nicely reproduce the energy and intensity differences encountered in the experimental data (Fig. 6).

## Conclusions

We previously reported that the 1-hole [4Cu:1S]^1−^ model cluster is oxidized to its 2-hole state by N_2_O with N_2_ evolution.^22^ Here, we have measured the reaction stoichiometry, allowing us to conclude that the overall 2-electron reduction of N_2_O requires two equivalents of the [4Cu:1S]^1−^ cluster molecule, with each equivalent mediating a 1-electron redox process individually. Under the assumption that one equivalent activates N_2_O while the other acts as a sacrificial reductant, a computational model of the reaction intermediates indicated cooperative Cu/S coordination of N_2_O.

This cooperative binding mode implies direct participation of the bridging S-atom in N_2_O activation and N–O cleavage, in contrast to the passive role of bridging S-atoms in typical metal–sulfur active sites. Consistent with this proposal, XAS analysis of the 1-hole and 2-hole clusters indicated that the μ_4_-S center contributes appreciably to the redox-active molecular orbital. Crucially, the S K-edge energies and estimated S-atom participation in redox chemistry closely match previous characterization of the biological Cu_Z_ site, making this synthetic system a faithful model in terms of electronic structure as well as atomic connectivity and chemical reactivity. Moreover, to our knowledge this data represents the first spectroscopic interrogation of multiple redox levels of a conserved [4Cu:1S] cluster.

Key to the model cluster's reactivity, and in particular to the μ_4_-S center's active participation in N_2_O activation and reduction, is the high degree of covalency within the [4Cu:1S] core. This Cu/S covalency allows the S-atom to exhibit characteristics typically associated with transition metals, such as the ability to simultaneously accept and donate electron density to/from the substrate and to vary its oxidation level during a chemical process, that are necessary for a catalytic active site mediating a multielectron redox process. Thus, it is important to consider both metal/metal and metal/ligand cooperation when interrogating highly covalent multinuclear catalysts such as Cu_Z_ and related systems.

## Conflicts of interest

There are no conflicts to declare.

## Supplementary Material

SC-011-C9SC06251C-s001

SC-011-C9SC06251C-s002
