# Supplementary material for: Probing the electronic and mechanistic roles of the μ4-sulfur atom in a synthetic CuZ model system
Source: Chem Sci. 2020 Feb 17;11(13):3441–7. doi: 10.1039/c9sc06251c (PMC8515425; doi:10.1039/c9sc06251c)
Supplement: SC-011-C9SC06251C-s001 [file SC-011-C9SC06251C-s001.pdf]

Electronic Supplementary Material (ESI) for Chemical Science.
This journal is © The Royal Society of Chemistry 2020

Probing the Electronic and Mechanistic Roles of the μ_4-Sulfur Atom in a Synthetic Cu_Z Model System

Suresh C. Rathnayaka,[a] Shahidul M. Islam,[a] Ida M. DiMucci,[b] Samantha N. MacMillan,[b] Kyle M. Lancaster,[b*] Neal P. Mankad[a*]

[a] Department of Chemistry, University of Illinois at Chicago, 845 W. Taylor St., Chicago, IL 60607
[b] Department of Chemistry & Chemical Biology, Cornell University, Baker Laboratory, Ithaca, NY 14853

*Corresponding Authors: npm@uic.edu and kml236@cornell.edu

Supporting information

Table of Contents

General Experimental Information	S2
Quantification of Produced N_2 by Headspace Analysis	S3
Computational Details for Optimization of Reactive Intermediate Structures	S21
Experimental Details for X-ray Spectroscopy	S37
Computational Details for X-ray Spectroscopy	S35
References	S41

General Experimental Information

All solvents except acetone were purchased from commercial suppliers, purified under argon with a Glass Contour Solvent System built by Pure Process Technology, and stored in a N_2-filled glovebox over 4-Å molecular sieves. Acetone (extra dry) was purchase from Acros and also treated with extra molecular sieves for further purification. Deuterated solvents were degassed and purified with 4-Å molecular sieves before use. All reactions were operated under N_2 with standard glovebox and Schlenk line techniques unless otherwise indicated. Medical grade nitrous oxide was purchased from Praxair and passed through a Drierite column for delivery to reaction vessels.
NMR spectra for compound characterization were recorded at ambient temperature using a Bruker Avance DPX-400 spectrometer. Chemical shifts are reported in ppm units relative to the residual signal of the solvent. Synthesis of the 1-hole and 2-hole [Cu_4S] clusters was based on literature procedures.[1] N_2 samples for calibration curves were syringed using a Hamilton gas tight syringe (10 µL, Model 1801 RN, Small Removable Needle, 26s gauge, 2 in, point style 2). Reaction and control head space gas samples (50 µL) were collected using Hamilton gas tight syringe (100 µL, Model 1710 SL SYR, customized Removable NDL(1 inch), 22s ga, 2 in, point style 2). GCMS data were collected using Agilent 5977B MSD sytem coupled to Agilent 7820A GC system with a CP-Molseieve 5A column (see Tables S3-S6 for other instrument, column and inlet control parameters). GCMS data analysis was performed using Agilent MassHunter Analysis Navigator B.08.00 software.

Quantification of Produced N_2 by Headspace Analysis of the Reaction between [Cu_4(μ_4-S)(μ_2-2,4,6-trimethylphenylformamidinate)$_4$][K(18-crown-6)] (1-hole) and N_2O in acetone.

Construction of a calibration curve: Five Schlenk flasks fitted with screw-cap septa (Headspace screwTin cap with PTFE/ butyl septum) were vacuum-refilled 3 times and filled with pure nitrogen. 2, 4, 6, 8 and 10 µL of pure N_2 samples were syringed separately from each flask and were injected into the GCMS. Peak area for each standard N_2 sample was recorded and plotted against the corresponding volume of N_2 to construct a calibration curve "Peak area Vs Volume of N_2 (µL)". Retention time for O_2 (trace) and N_2 are 4.3 and 6.1 min respectively.

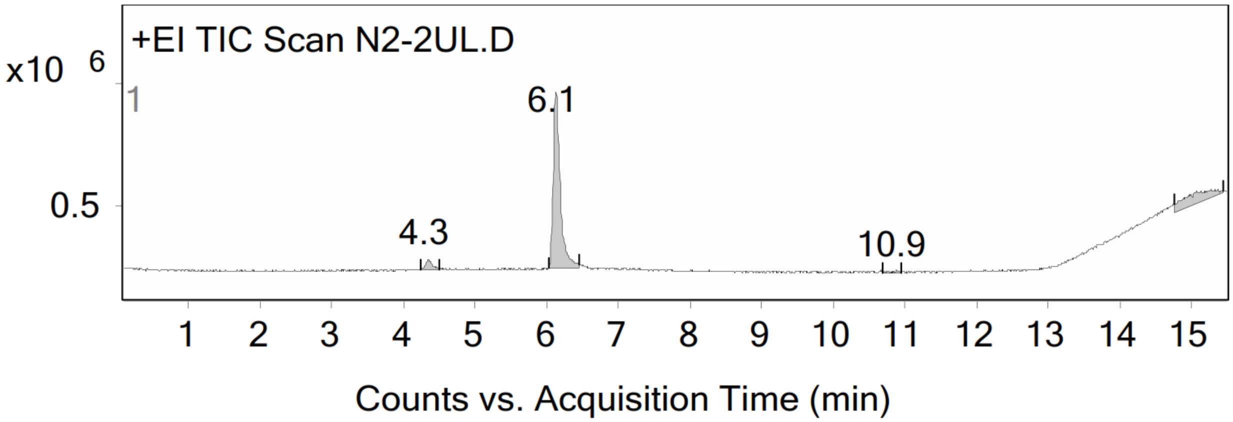

Integration Peak List

Peak	Start	RT	End	Height	Area	Area %
1	4.2	4.3	4.5	41590.73	263341.39	5.3
2	6	6.1	6.5	718399.34	4965640.6	100
3	10.7	10.9	10.9	9736.76	65628.54	1.32
4	14.8	15.3	15.4	31036.05	1306016.5	26.3

Figure S1. The Total Ion Chromatogram (TIC) and the peak integration for 2 µL N_2 standard.

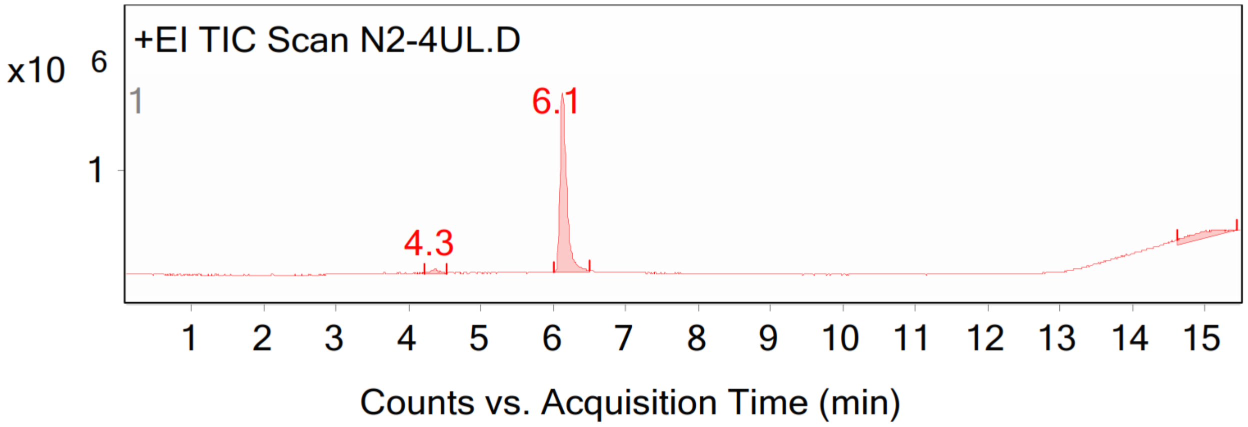

Integration Peak List

Peak	Start	RT	End	Height	Area	Area %
1	4.2	4.3	4.5	29764.15	238490.78	2.72
2	6	6.1	6.5	1327530.52	8755552.42	100
3	14.6	15.2	15.4	41961.67	1804931.24	20.61

Figure S2. The Total Ion Chromatogram (TIC) and the peak integration for 4 μL N_2 standard.

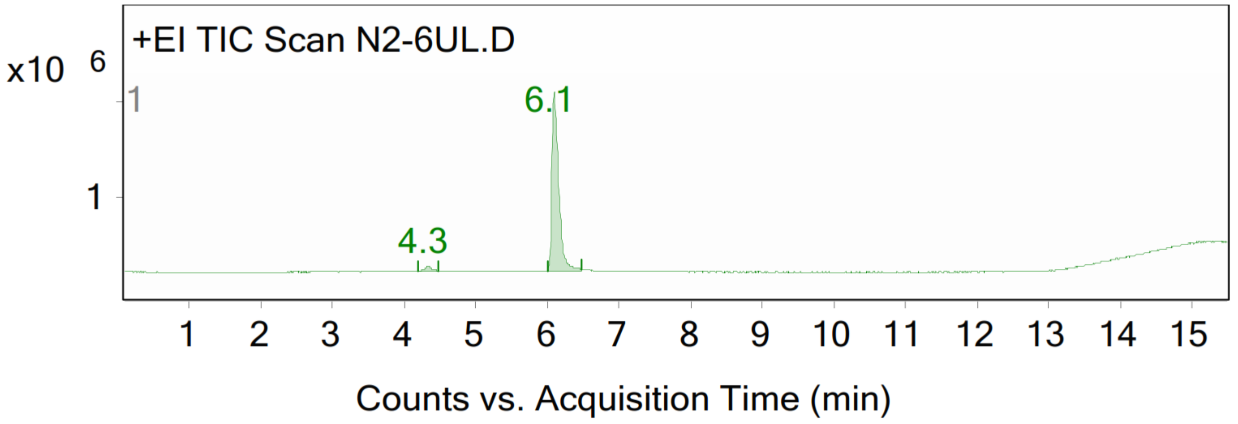

Integration Peak List

Peak	Start	RT	End	Height	Area	Area %
1	4.2	4.3	4.5	52690.52	373016.63	3.08
2	6	6.1	6.5	1858441.63	12117721.07	100

Figure S3. The Total Ion Chromatogram (TIC) and the peak integration for 6 μL N_2 standard.

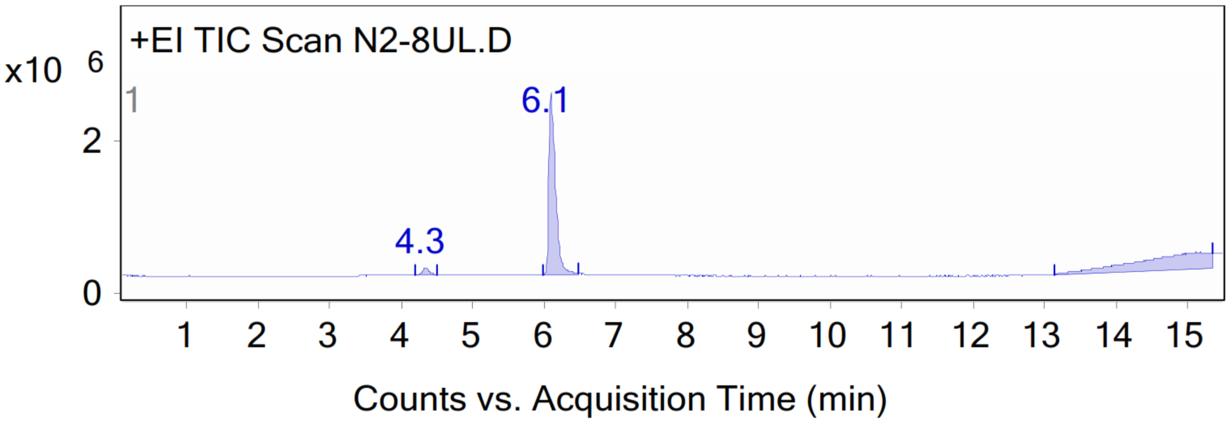

Integration Peak List

Peak	Start	RT	End	Height	Area	Area %
1	4.2	4.3	4.5	93396.29	647698.25	4.13
2	6	6.1	6.5	2403337.82	15482161.84	98.64
3	13.1	15.2	15.3	219968.1	15696028.35	100

Figure S4. The Total Ion Chromatogram (TIC) and the peak integration for 8 μL N_2 standard.

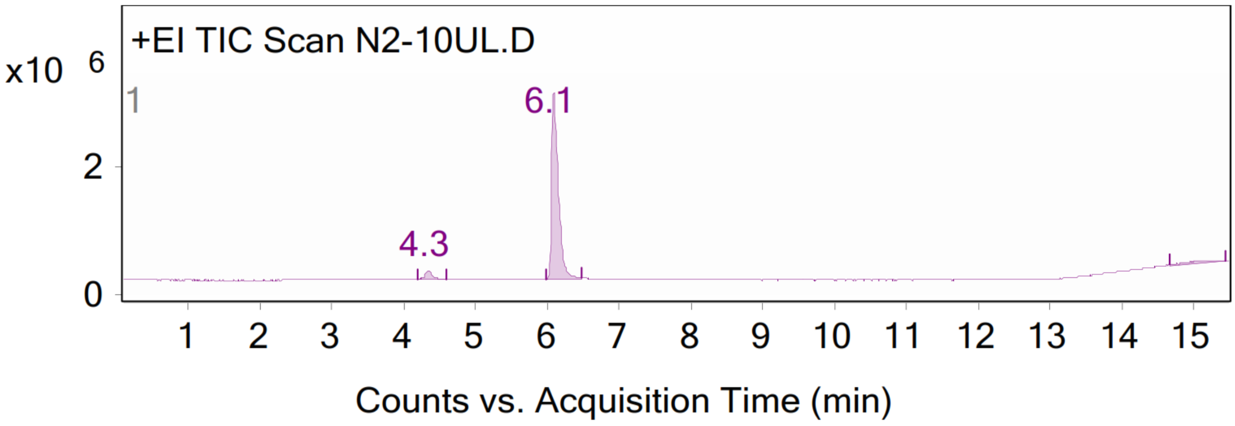

Integration Peak List

Peak	Start	RT	End	Height	Area	Area %
1	4.2	4.3	4.6	141987.09	970282.76	5.12
2	6	6.1	6.5	2914764.09	18934075.64	100
3	14.7	15.2	15.4	33154.09	1445020.29	7.63

Figure S5. The Total Ion Chromatogram (TIC) and the peak integration for 10 μL N_2 standard.

Headspace analysis of the reaction:

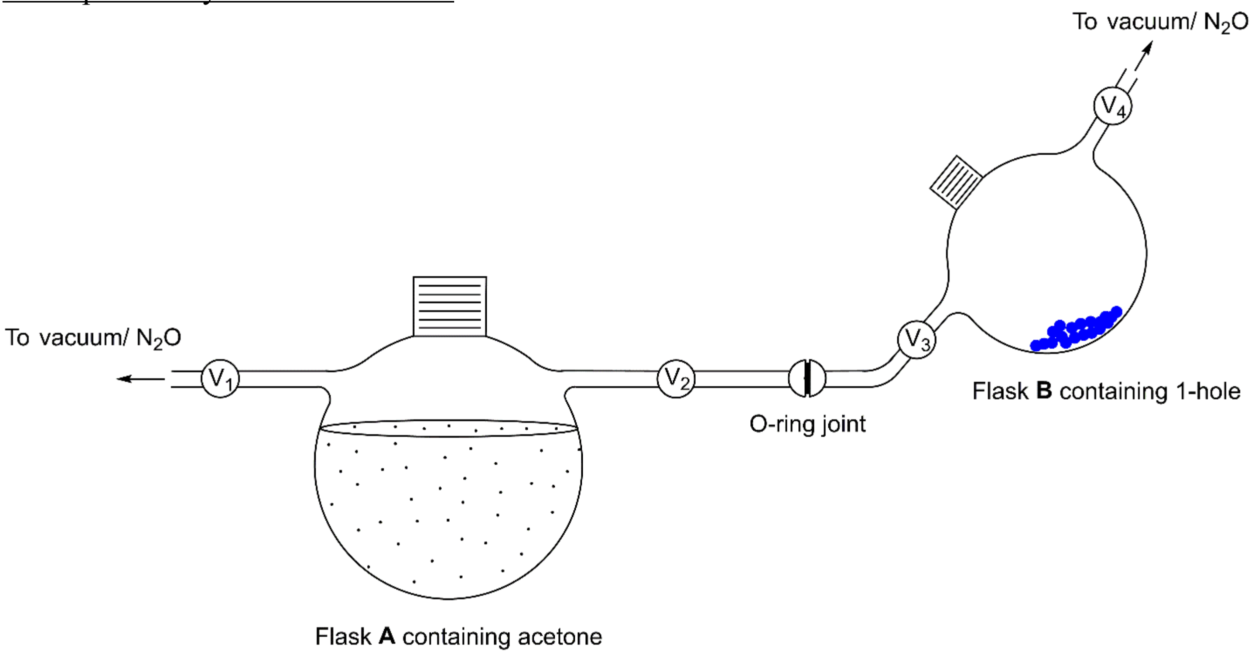

Figure S6. Experimental setup used for headspace analysis.

The apparatus shown above (Figure S6) was used for the analysis to minimize any background N_2 and to avoid any air oxidation of the 1-hole cluster. In a N_2 filled glove box, flask **A** was charged with 55.0 mL of acetone and a magnetic stir bar. Similarly, flask **B** was charged with [Cu_4(μ_4-S)(μ_2-2,4,6-trimethylphenylformamidinate)$_4$][K(18-crown-6)] (1-hole) (148.9 mg, 0.087 mmol) and a magnetic stir bar. Both flasks were secured with screw-cap septa (Headspace screwTin cap with PTFE/ butyl septum), and then flask **B** was then connected to a vacuum line and evacuated for 30 min. Both flasks were taken out of the glove box after closing the valves V_1, V_2, V_3 and V_4. Flasks were connected at the O-ring joint and were attached to Schlenk line (N_2O/vacuum) at V_1 and V_4. Acetone in flask **A** was frozen using liquid nitrogen and the headspace was evacuated by opening valves V_1, V_2, V_3 and V_4 to vacuum for 5 min. Next, V_1 and V_4 were closed and the acetone was allowed to thaw. During the thawing process, V_4 was opened to vacuum (for 3 sec) occasionally to release any buildup pressure. Then V_2 was closed and acetone in flask **A** was frozen again while keeping V_4 open to vacuum. Similarly, two more freeze-pump-thaw cycles were performed to remove remaining headspace gases and any dissolved gases. After the final cycle, acetone was allowed to reach room temperature for 20 min and V_2 was closed and V_4 was opened to vacuum. Headspace of Flask **A** was filled with N_2O by opening V_1 to N_2O. V_1 was then closed and the acetone was stirred for 30 min allowing N_2O to equilibrate. Then, the space between V_2 and V_4 was filled with N_2O by opening V_4 to N_2O. Finally, V_4 was closed and the acetone in flask **A** was transferred into flask **B** by tilting the entire setup clockwise, while keeping V_1 open to N_2O. Once all the acetone was transferred, V_1 and V_3 were closed and the flask **B** was detached at the O-ring and from the Schlenk line. The reaction mixture was stirred for 4 h at -78 ^oC and allowed to reach room temperature over 1 h. A 50-µL portion of the headspace was syringed (after flushing the needle 3 times with Ar) and analyzed using GCMS. Retention times for Ar, N_2 and N_2O were 4.3, 6.1 and 14.9 min respectively. H_2O from the CP-molsieve 5A column began to elute around

12.3 min. The reaction mixture was taken back into the glove box and filtered through a fine frit to recover the produced 2-hole. Solid on the frit was rinsed with 2×10 mL of acetone and 10 mL of Et_2O. The dark residue was completely dried under vacuum and NMR was taken in CD_2Cl_2 (**Figure S13**). Filtrate was completely evaporated, and NMR was taken in acetone-d_6 (**Figure S14**). A separate control experiment was carried out in the absence of 1-hole and the head space was analyzed in a similar manner. The whole experiment (both exp and control) was repeated two more times and the results were averaged.

Caution: With the limited head space volume, the thawing process must be done carefully while opening the head space to vacuum occasionally to prevent the Schlenk flask/ setup from exploding.

Table S1. Details of experiment and control runs of headspace analysis.

Parameter		Experiment	Control
1-hole		148.9 mg	-
Reaction gas		N_2O	N_2O
Total volume of reaction flask **B**		65.7 mL	65.5 mL
Total volume of acetone		55.0 mL	54.8 mL
Volume of Headspace		10.7 mL	10.7 mL
Recovered 2-hole	Trial 1	0.0477 g	N/A
	Trial 2	0.0679 g	
	Trial 2	0.0507 g	

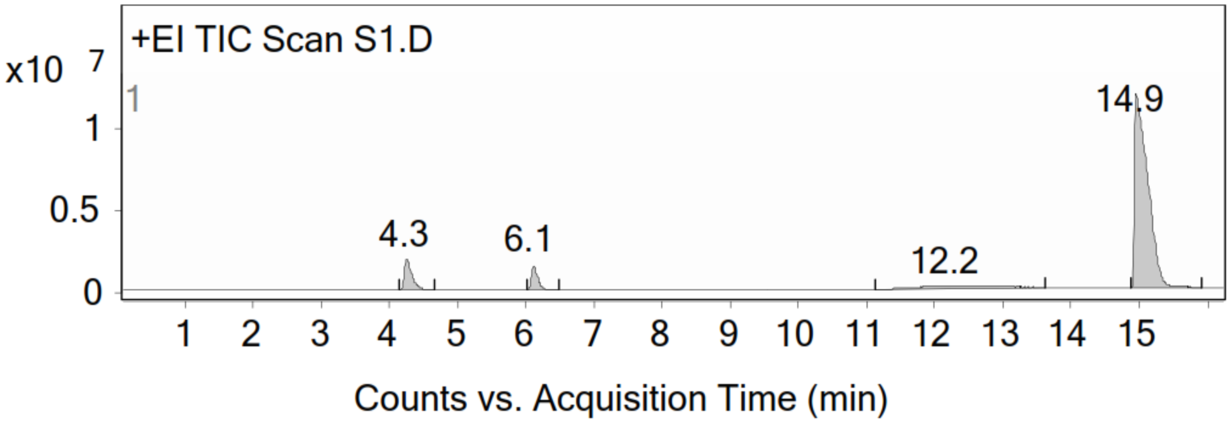

Integration Peak List

Peak	Start	RT	End	Height	Area	Area %
1	4.1	4.3	4.7	1854138.41	14809365.81	9.35
2	6	6.1	6.5	1480061.6	9328899.21	5.89
3	11.1	12.2	13.6	136005.82	11511281.87	7.27
4	14.9	14.9	15.9	11791901.95	158371163.73	100

Figure S7. Total Ion Chromatogram (TIC) and the peak integration of the headspace (50 µL) from the reaction between 1-hole and N_2O in acetone – Trial 1

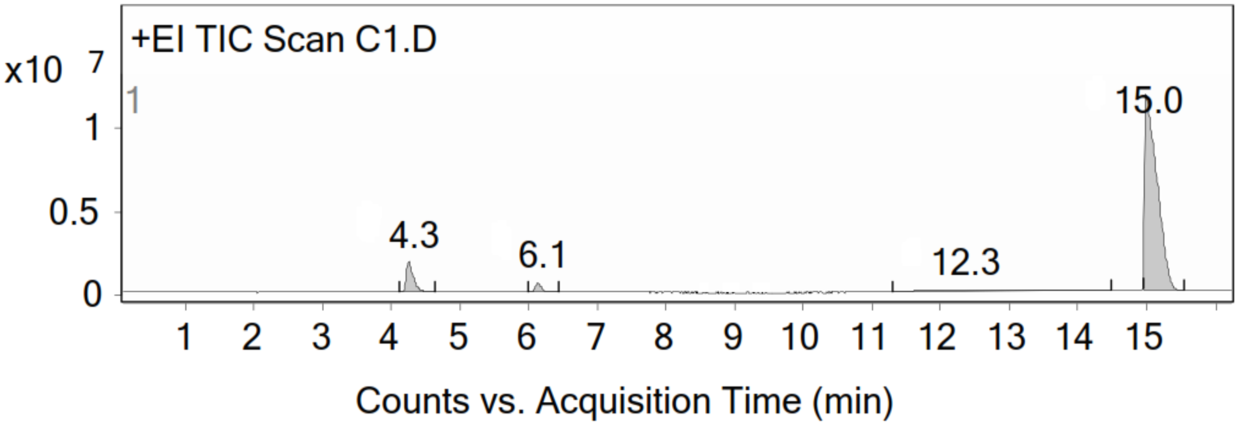

Integration Peak List

Peak	Start	RT	End	Height	Area	Area %
1	4.1	4.3	4.6	1760896.17	13580475.57	9.18
2	6	6.1	6.4	592103.9	3701271.78	2.5
3	11.3	12.3	14.5	117155.62	11468083.8	7.76
4	14.9	15	15.6	11708257.9	147877861.22	100

Figure S8. Total Ion Chromatogram (TIC) and the peak integration of the headspace (50 µL) from the control reaction – Trial 1

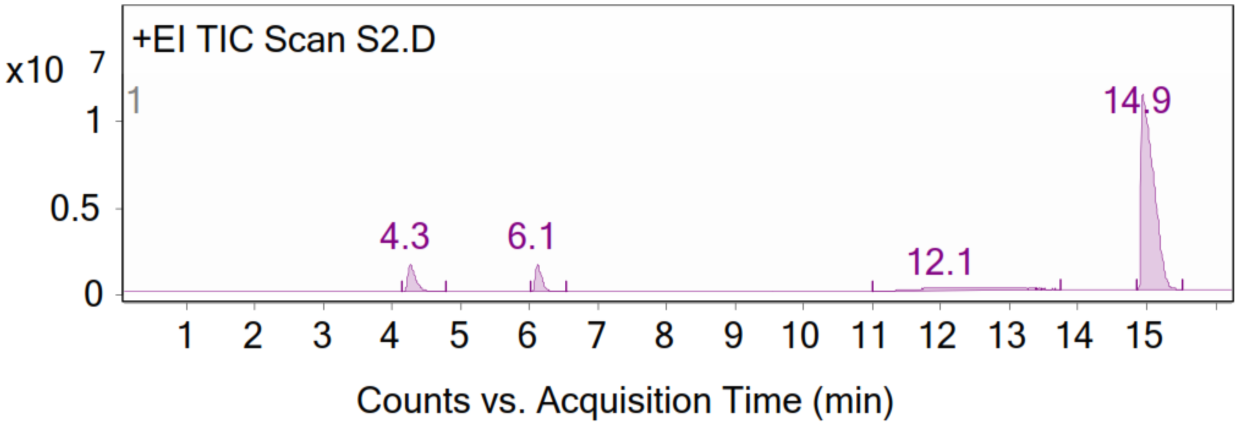

Integration Peak List

Peak	Start	RT	End	Height	Area	Area %
1	4.2	4.3	4.8	1619286.58	13022682.39	9.23
2	6	6.1	6.5	1620249.44	10470446	7.42
3	11	12.1	13.7	142052.64	12330752.89	8.74
4	14.9	14.9	15.5	11285538.93	141150950.34	100

Figure S9. Total Ion Chromatogram (TIC) and the peak integration of the headspace (50 µL) from the reaction between 1-hole and N_2O in acetone – Trial 2

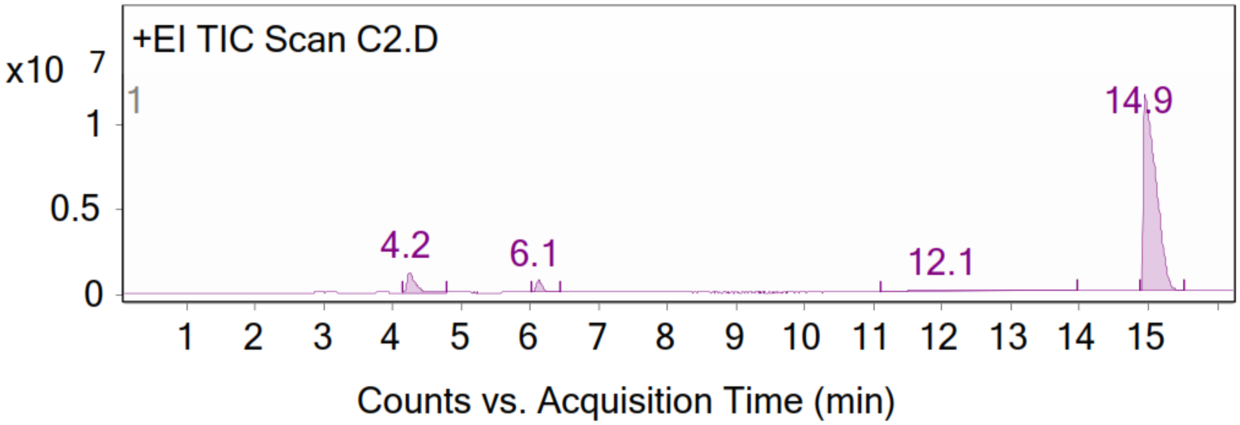

Integration Peak List

Peak	Start	RT	End	Height	Area	Area %
1	4.1	4.2	4.8	1154788.66	9846659.58	6.9
2	6	6.1	6.4	706874.39	4343091.95	3.04
3	11.1	12.1	14	128114.95	11585254.9	8.11
4	14.9	14.9	15.5	11455672.39	142771694.92	100

Figure S10. Total Ion Chromatogram (TIC) and the peak integration of the headspace (50 µL) from the control reaction – Trial 2

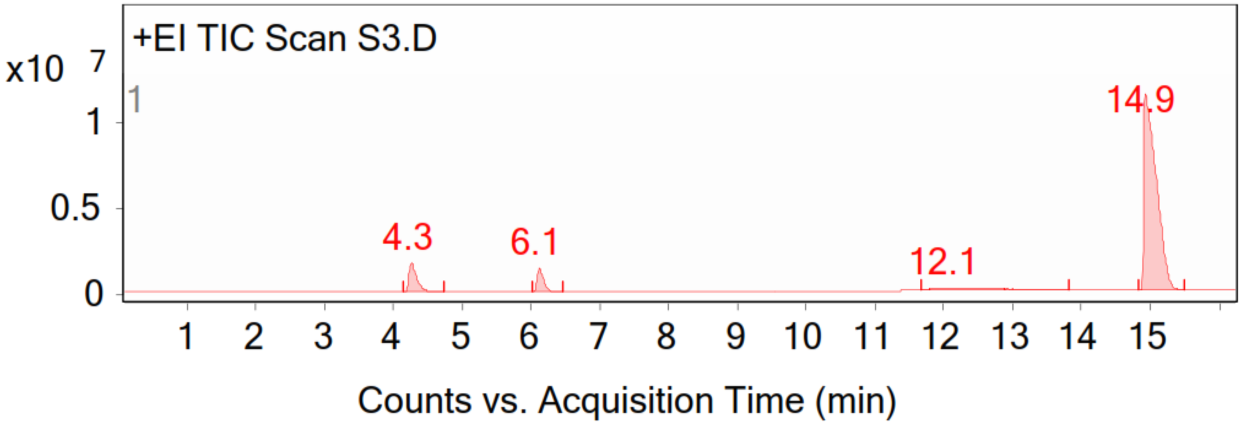

Integration Peak List

Peak	Start	RT	End	Height	Area	Area %
1	4.1	4.3	4.7	1692061.81	13930292.04	9.79
2	6	6.1	6.5	1328445.76	8994435.01	6.32
3	11.7	12.1	13.8	51318.33	3433487.06	2.41
4	14.8	14.9	15.5	11321683.11	142288175.79	100

Figure S11. Total Ion Chromatogram (TIC) and the peak integration of the headspace (50 µL) from the reaction between 1-hole and N_2O in acetone – Trial 3

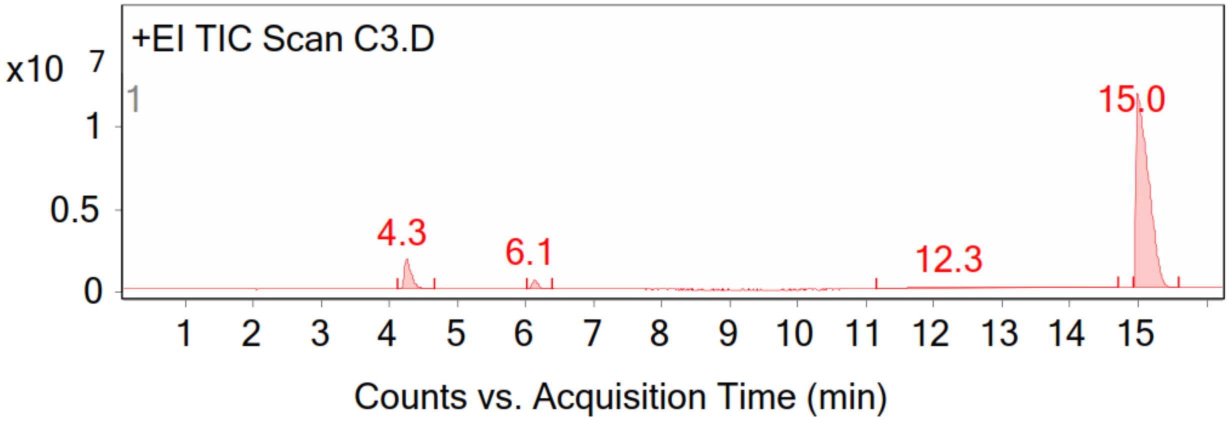

Integration Peak List

Peak	Start	RT	End	Height	Area	Area %
1	4.1	4.3	4.7	1765110.59	13859762.11	9.3
2	6	6.1	6.4	590501.95	3657080.5	2.45
3	11.2	12.3	14.7	129757.5	13863590.69	9.3
4	14.9	15	15.6	11759648.25	148998148.69	100

Figure S12. Total Ion Chromatogram (TIC) and the peak integration of the headspace (50 µL) from the control reaction – Trial 3

Data analysis:

Table S2. N_2 peak area for standards (2-10 μL), Controls and experiments.

Sample	N_2 peak area (integration)	
Standard 2 μL	4,965,640	
Standard 4 μL	8,755,552	
Standard 6 μL	12,117,721	
Standard 8 μL	15,482,161	
Standard 10 μL	18,934,075	
	Experiment	Control
Head space trial 1	9,328,899	3,701,271
Head space trial 2	10,470,446	4,343,091
Head space trial 3	8,994,435	3,657,080

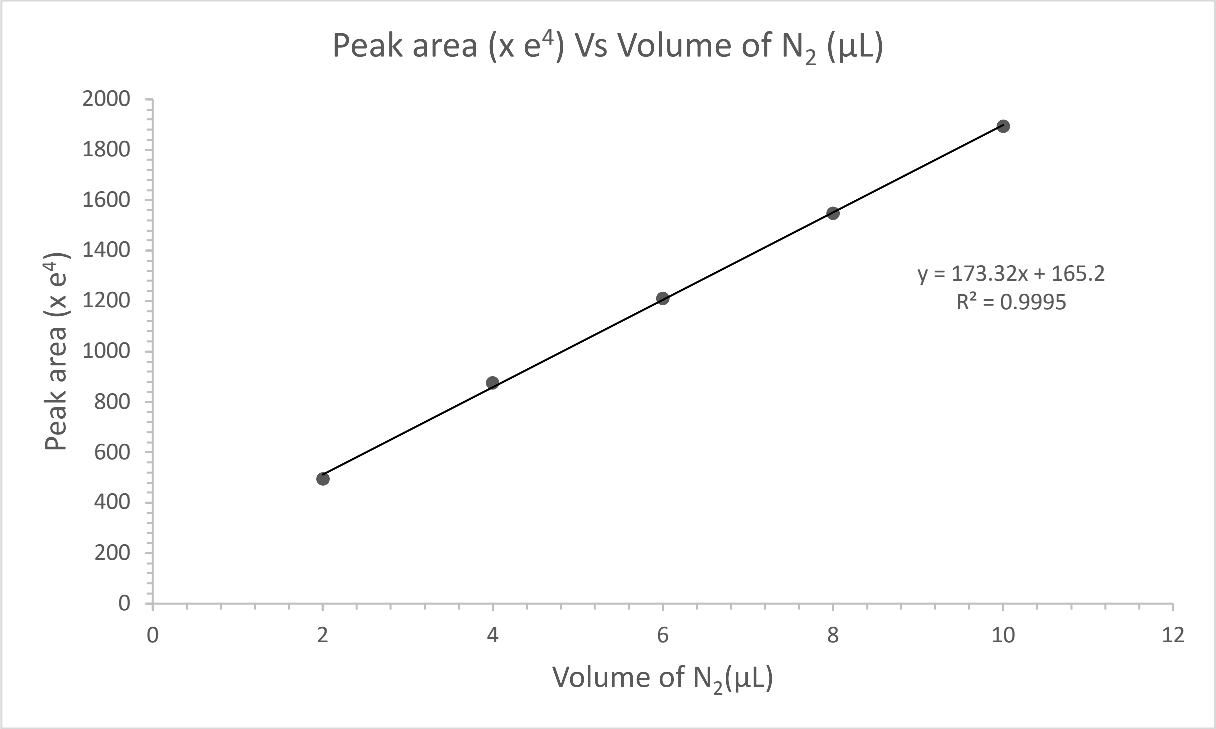

Figure S13. The calibration curve "Peak area (x e^4) Vs volume of N_2 (μL)

Calculation for produced N_2:

Assumptions:
1. Room temperature and pressure do not change significantly over the course of entire analysis.
2. N_2 behaves as an ideal gas

Calculations for headspace exp 1:

Moles of 1-hole complex $= \frac{1\ mol}{1707.29\ g} \times 0.1489\ g$

$= 0.087\ mmol$

N_2 peak area for the experiment $= 9{,}328{,}899$

N_2 peak are for the control experiment $= 3{,}701{,}271$

Peak area for produced N_2 from the reaction $= 9{,}328{,}899 - 3{,}701{,}271$

$= 5{,}627{,}628$

Produced N_2 volume in 50 µL of the headspace (by interpolating from the calibration curve) $= 2.29\ \mu L$

Produced N_2 volume in total 10.7 mL of headspace $= \frac{2.29\ \mu L}{50\ \mu L} \times 10.7\ mL$

$= 0.49\ mL$

Produced N_2 moles from the reaction (using PV = nRT) $= \frac{PV}{RT}$

$$= \frac{1\ atm \times 0.49 \times 10^{-3}\ L}{0.082\ L.atm.K^{-1}.mol^{-1} \times 298\ K}$$

$= 0.020\ mmol$

Recovered 2-hole weight $= 0.0477\ g$

Produced 2-hole $= 0.034\ mmol$

Consumed 1-hole $= 0.034\ mmol$

1-hole : N_2 $= 0.034 : 0.020$

$= 1 : 0.59$

Yield of 2-hole = 39 %

Calculations for headspace exp 2:

Moles of 1-hole $= \frac{1\ mol}{1707.29\ g} \times 0.1489\ g$

$= 0.087\ mmol$

N_2 peak area for the experiment $= 10{,}470{,}446$

N_2 peak are for the control experiment $= 4{,}343{,}091$

Peak area for produced N_2 from the reaction $= 10{,}470{,}446 - 4{,}343{,}091$

$= 6{,}127{,}355$

Produced N_2 volume in 50 µL of the headspace (by interpolating from the calibration curve) $= 2.58\ \mu L$

Produced N_2 volume in total 10.7 mL of headspace $= \frac{2.58\ \mu L}{50\ \mu L} \times 10.7\ mL$

$= 0.55 mL$

Produced N_2 moles from the reaction (using PV = nRT) $= \frac{PV}{RT}$

$$= \frac{1\ atm \times 0.55 \times 10^{-3}\ L}{0.082\ L.atm.K^{-1}.mol^{-1} \times 298\ K}$$

$= 0.022\ mmol$

Recovered 2-hole weight $= 0.0642\ g$

Produced 2-hole $= 0.046\ mmol$

Consumed 1-hole $= 0.046\ mmol$

1-hole : N_2 $= 0.046 : 0.022$

$= 1 : 0.48$

Yield of 2-hole = 53 %

Calculations for headspace exp 3:

Moles of 1-hole $= \frac{1\ mol}{1707.29\ g} \times 0.1489\ g$

$= 0.087\ mmol$

N_2 peak area for the experiment $= 8{,}994{,}435$

N_2 peak are for the control experiment $= 3{,}657{,}080$

Peak area for produced N_2 from the reaction $= 8{,}994{,}435 - 3{,}657{,}080$

$= 5{,}337{,}355$

Produced N_2 volume in 50 μL of the headspace (by interpolating from the calibration curve) $= 2.13\ \mu L$

Produced N_2 volume in total 10.7 mL of headspace $= \frac{2.13\ \mu L}{50\ \mu L} \times 10.7\ mL$

$= 0.46 mL$

Produced N_2 moles from the reaction (using PV = nRT) $= \frac{PV}{RT}$

$$= \frac{1\ atm \times 0.46 \times 10^{-3}\ L}{0.082\ L.atm.K^{-1}.mol^{-1} \times 298\ K}$$

$= 0.019\ mmol$

Recovered 2-hole weight $= 0.0507\ g$

Produced 2-hole $= 0.036\ mmol$

Consumed 1-hole $= 0.036\ mmol$

1-hole : N_2 $= 0.036 : 0.019$

$= 1 : 0.52$

Yield of 2-hole = 41 %

Average N_2 moles produced per 1 mole of 1-hole $= 0.53\ mol$

Standard deviation $= \sqrt{\frac{\sum_{i=1}^{3}(x_i - \bar{x})^2}{n-1}}$

x_i = N_2 moles per 1 mole of 1-hole for the ith headspace exp

$\bar{x}$ = Average N_2 moles produced per 1 mole of 1-hole

n = 3

$= \pm\ 0.06$

Produced N_2 moles per 1 mole of 1-hole $= 0.53\ (\pm 0.06)\ mol$

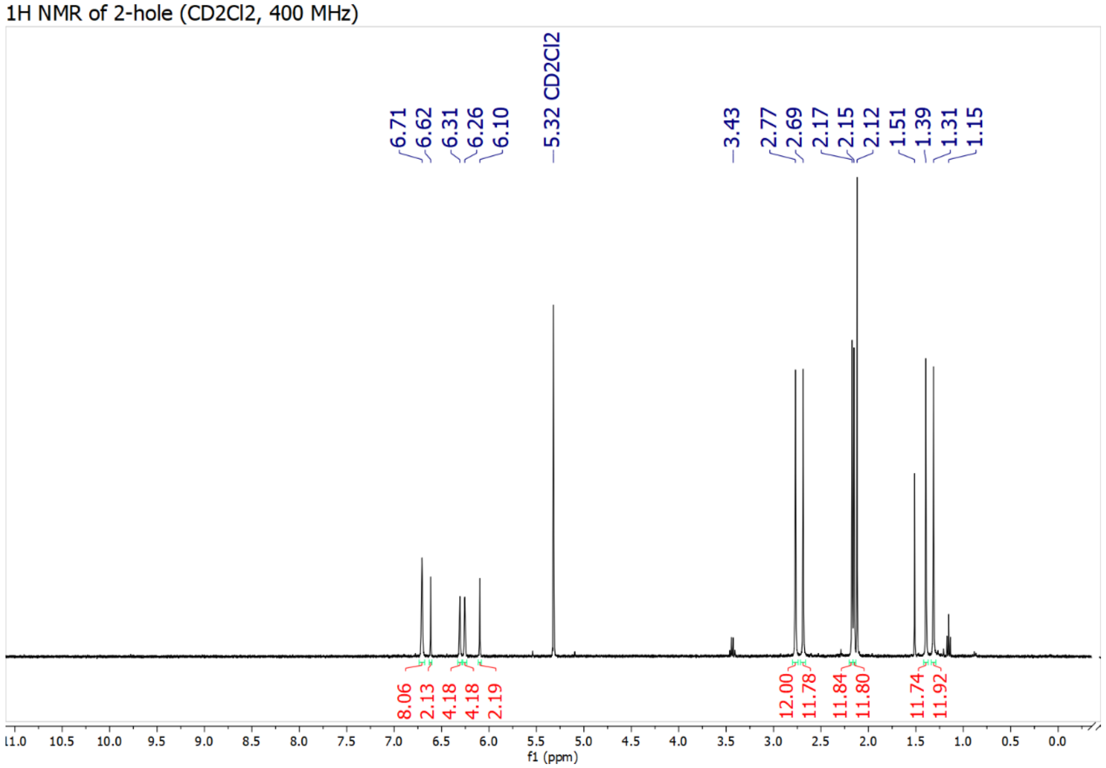

Figure S14. Sample NMR of the 2-hole recovered. ^{1}H NMR (400 MHz, CD_2Cl_2) δ 6.71 (s, 8H, Ar CH), 6.62 (s, 2H, NC(H)N), 6.31 (s, 4H, Ar CH), 6.26 (s, 4H, Ar CH), 6.10 (s, 2H, NC(H)N), 2.77 (s, 12H, Ar CH3), 2.69 (s, 12H, Ar CH3), 2.17 (s, 12H, Ar CH3), 2.15 (s, 12H, Ar CH3), 2.12 (s, residual acetone), 1.51 (s, residual H_2O), 1.39 (s, 12H, Ar CH3), 1.31 (s, 12H, Ar CH3), 3.43 (q, residual Et_2O), 1.15 (t, residual Et_2O)

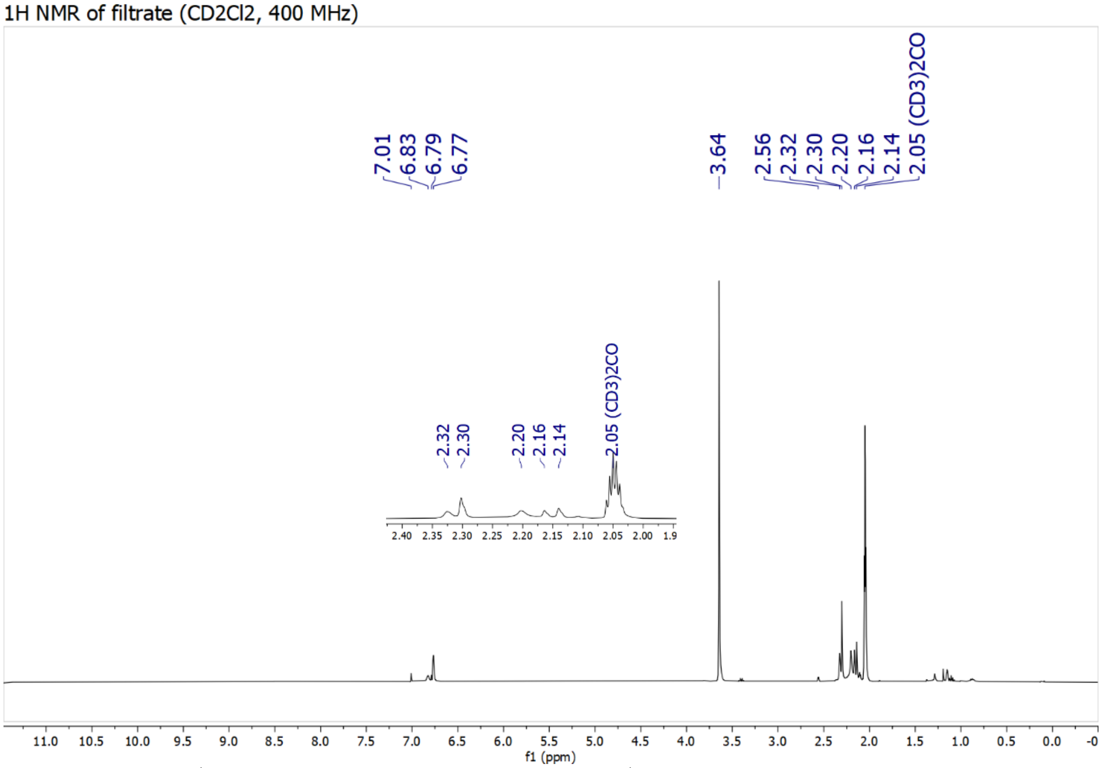

Figure S15. Sample ^{1}H NMR of the filtrate collected. ^{1}H NMR (400 MHz, CD_2Cl_2) δ 3.64 (s, 18-crown-6), 2.05 (aceton-d_6). Characteristic peaks for $Cu_2(NCN)_2$ precursor were located at δ 7.01, 6.79, 2.30 and 2.20 overlapping with unidentified byproducts.

Table S3. MS parameter report for calibration.

Single Quadrupole Acquisition Method - MS Parameters Report

**Method file**	D:\MassHunter\GCMS\1\methods\SureshNitrogent.m
**Tune file**	ATUNE.U
**Ion source**	EI
**Source temperature (°C)**	250
**Quad temperature (°C)**	150
**Fixed Electron energy (eV)**	70.0
**Acquisition Type**	Scan
**Stop time (min)**	650.00
**Solvent delay (min)**	0.00
**Trace Ion Detection**	False
**Gain Factor**	1
**EM Saver**	False
**EM Saver Limit**	N/A

Scan Time Segments

Time	Start Mass	End Mass	Threshold	Scan Speed
0.00	9	839	150	1,562 [N=2]
15.50	9	839	150	1,562 [N=2]

Timed Events

Time	Type of Event	Parameter

Real-Time Plots

Type of Plot	Label	Low Mass	High Mass
Spectrum	N/A	N/A	N/A
Base Peak	N/A	N/A	N/A
Extracted Ion	Scan 1-1	9	839

Self-Cleaning Ion Source Parameters

Mode	No Cleaning

Table S4. Instrumental control parameters for standard samples (calibration).

```
INSTRUMENT CONTROL PARAMETERS:    Agilent 5977B MSD System
----------------------------------------------------------

D:\MassHunter\GCMS\1\methods\SureshNitrogent.m
   Thu Nov 21 12:13:57 2019

Control Information
------- -----------

Sample Inlet            : GC
Injection Source        : Manual
Mass Spectrometer       : Enabled

No Sample Prep method has been assigned to this method.

GC
GC Summary
Run Time                                  15.5 min
Post Run Time                             3 min

Oven
Equilibration Time                        0.2 min
Max Temperature                           320 °C
Maximum Temperature Override              Disabled
Slow Fan                                  Disabled
Temperature
Setpoint                                  On
(Initial)                                 50 °C
Hold Time                                 2 min
Post Run                                  50 °C
Program
#1 Rate                                   20 °C/min
#1 Value                                  300 °C
#1 Hold Time                              1 min

Front SSZ Inlet He
Mode                                      Split
Heater                                    On    250 °C
Pressure                                  On    1.2 psi
Gas Saver                                 Off
Split Ratio                               20 :1
Split Flow                                4.0835 mL/min

Thermal Aux 1 (MSD Transfer Line)
Temperature
Setpoint                                  On
(Initial)                                 300 °C
Post Run                                  0 °C

Column
Column Outlet Pressure                    0 psi
Column #1
Column Information                        Agilent CP7533
CP-Molsieve 5Å
Temperature Range                         -60 °C—350 °C (350 °C)
Dimensions                                25 m x 250 µm x 30 µm
In                                        Front SSZ Inlet He
Out                                       MSD
(Initial)                                 50 °C

Pressure                                  1.2 psi
Flow                                      0.20417 mL/min
Average Velocity                          18.04 cm/sec
Holdup Time                               2.3097 min
Flow
Setpoint                                  Off
(Initial)                                 0.20417 mL/min
Post Run                                  1 mL/min
```

Table S5. MS parameter report for N_2 quantification experiments and controls.

Single Quadrupole Acquisition Method - MS Parameters Report

**Method file**	D:\MassHunter\GCMS\1\methods\SureshN2O.m
**Tune file**	ATUNE.U
**Ion source**	EI
**Source temperature (°C)**	250
**Quad temperature (°C)**	150
**Fixed Electron energy (eV)**	70.0
**Acquisition Type**	Scan
**Stop time (min)**	650.00
**Solvent delay (min)**	0.00
**Trace Ion Detection**	False
**Gain Factor**	1
**EM Saver**	False
**EM Saver Limit**	N/A

Scan Time Segments

Time	Start Mass	End Mass	Threshold	Scan Speed
0.00	9	839	150	1,562 [N=2]
15.50	9	839	150	1,562 [N=2]

Timed Events

Time	Type of Event	Parameter

Real-Time Plots

Type of Plot	Label	Low Mass	High Mass
Spectrum	N/A	N/A	N/A
Base Peak	N/A	N/A	N/A
Extracted Ion	Scan 1-1	9	839

Self-Cleaning Ion Source Parameters

Mode	No Cleaning

Table S6. Instrumental control parameters for N_2 quantification experiments and controls.

```
            INSTRUMENT CONTROL PARAMETERS:    Agilent 5977B MSD System
            --------------------------------------------------------

    D:\MassHunter\GCMS\1\methods\SureshN2O.m
       Thu Nov 21 12:17:10 2019

 Control Information
 ------- -----------

 Sample Inlet              : GC
 Injection Source          : Manual
 Mass Spectrometer         : Enabled

  No Sample Prep method has been assigned to this method.

 GC
 GC Summary
 Run Time                                     16.25 min
 Post Run Time                                3 min

 Oven
 Equilibration Time                           0.2 min
 Max Temperature                              320 °C
 Maximum Temperature Override                 Disabled
 Slow Fan                                     Disabled
 Temperature
 Setpoint                                     On
 (Initial)                                    50 °C
 Hold Time                                    2 min
 Post Run                                     50 °C
 Program
 #1 Rate                                      20 °C/min
 #1 Value                                     150 °C
 #1 Hold Time                                 0 min
 #2 Rate                                      40 °C/min
 #2 Value                                     300 °C
 #2 Hold Time                                 5.5 min


 Front SSZ Inlet He
 Mode                                         Split
 Heater                                       On    250 °C
 Pressure                                     On    1.2 psi
 Gas Saver                                    Off
 Split Ratio                                  20 :1
 Split Flow                                   4.0835 mL/min

 Thermal Aux 1 (MSD Transfer Line)
 Temperature
 Setpoint                                     On
 (Initial)                                    300 °C
 Post Run                                     0 °C


 Column
 Column Outlet Pressure                       0 psi
 Column #1
 Column Information                           Agilent CP7533
 CP-Molsieve 5Å
 Temperature Range                            -60 °C—350 °C (350 °C)
 Dimensions                                   25 m x 250 µm x 30 µm

 In                                           Front SSZ Inlet He
 Out                                          MSD
 (Initial)                                    50 °C
 Pressure                                     1.2 psi
 Flow                                         0.20417 mL/min
 Average Velocity                             18.04 cm/sec
 Holdup Time                                  2.3097 min
 Flow
 Setpoint                                     Off
 (Initial)                                    0.20417 mL/min
 Post Run                                     1 mL/min
```

Computational Details for Optimization of Reactive Intermediate Structures

All the electronic structure calculations were carried out with Gaussian16.[2] The geometries were fully optimized at the B3LYP level of theory using the 6-31G(d) basis set. Frequencies were calculated for all structures to ensure the absence of imaginary frequencies for energy minima. The optimized XYZ coordinates are provided below.

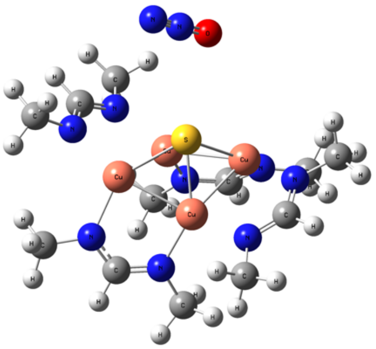

$[Cu_4S(NCN)_4]^-$ + N_2O reactant (Sum of electronic and thermal Free Energies= -8056.123923 Hartrees)

Center	Atomic	Atomic	Coordinates (Angstroms)		
Number	Number	Type	X	Y	Z
1	29	0	1.657760	-1.006228	-0.145533
2	16	0	-0.084767	-0.420511	-1.511342
3	7	0	3.310365	-0.570623	-1.173112
4	7	0	2.637751	1.670610	-1.179631
5	6	0	3.468181	0.697096	-1.504028
6	1	0	4.365554	0.962465	-2.095663
7	29	0	-1.573370	1.090212	-0.665257
8	7	0	-3.351214	0.189890	-0.675782
9	7	0	-2.485437	-1.442186	0.760724
10	6	0	-3.456923	-0.914550	0.038126
11	1	0	-4.433547	-1.435786	0.030363
12	29	0	-0.591586	-0.721877	0.656028
13	7	0	0.110119	-0.776851	2.473078
14	7	0	2.111394	-1.627766	1.635654
15	6	0	1.323805	-1.265286	2.634628
16	1	0	1.705495	-1.378955	3.665160

17	29	0	0.815173	1.283646	-0.385236
18	7	0	0.480304	2.714408	0.891126
19	7	0	-1.717299	2.857983	0.127375
20	6	0	-0.715297	3.269483	0.887202
21	1	0	-0.890675	4.130383	1.557014
22	6	0	-4.474344	0.540966	-1.516515
23	1	0	-4.770990	1.589668	-1.364395
24	1	0	-4.231649	0.432397	-2.586012
25	1	0	-3.834752	-2.985952	1.344666
26	1	0	-2.112820	-3.430170	1.353893
27	1	0	-2.761271	-2.401468	2.631856
28	6	0	-0.600073	-0.324481	3.649024
29	1	0	-0.709951	0.770833	3.650969
30	1	0	-1.613981	-0.745544	3.680326
31	1	0	-0.088593	-0.609423	4.584941
32	6	0	3.412187	-2.163927	1.982197
33	1	0	4.214427	-1.565094	1.529013
34	1	0	3.583645	-2.183372	3.072585
35	1	0	3.532552	-3.194997	1.614517
36	6	0	4.266847	-1.516503	-1.701493
37	1	0	5.112646	-1.024840	-2.215098
38	1	0	4.689937	-2.144759	-0.902715
39	1	0	3.800820	-2.202136	-2.427972
40	6	0	2.987608	3.004675	-1.619474
41	1	0	3.087641	3.697022	-0.769339
42	1	0	3.940654	3.030655	-2.177325
43	1	0	2.211381	3.424852	-2.276628
44	6	0	1.445109	3.190715	1.858053
45	1	0	1.635685	2.434880	2.635403
46	1	0	2.409162	3.400358	1.376260
47	1	0	1.112872	4.113691	2.364529
48	6	0	-2.961668	3.594258	0.223168
49	1	0	-2.911799	4.412849	0.962176
50	1	0	-3.238864	4.043810	-0.743382
51	1	0	-3.786355	2.930149	0.516916
52	7	0	-1.464493	-3.606168	-1.715601
53	7	0	-2.235795	-3.240037	-2.460056
54	8	0	-0.659023	-4.023242	-0.941303

55	1	0	-5.363410	-0.084734	-1.320375
56	6	0	-2.816524	-2.609833	1.551403

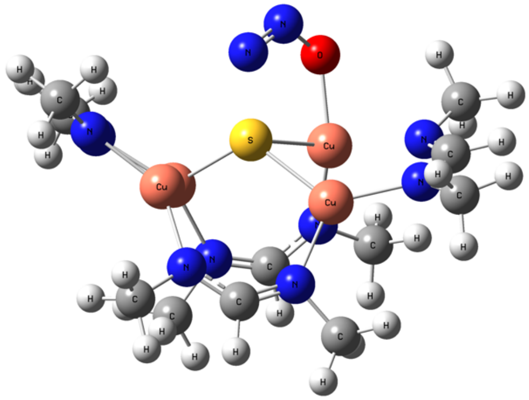

[$Cu_4S(NCN)_4 \cdot N_2O$]$^-$ (Sum of electronic and thermal Free Energies= -8056.089054 Hartrees)

Center Number	Atomic Number	Atomic Type	Coordinates (Angstroms) X	Y	Z
1	29	0	1.748073	-1.200321	0.053598
2	16	0	-0.166276	-0.379237	-0.886533
3	7	0	3.681464	-0.937406	-0.352092
4	7	0	3.202753	1.220786	-1.134240
5	6	0	4.006638	0.183694	-0.967578
6	1	0	5.030874	0.260451	-1.374708
7	29	0	-2.206976	0.825488	-0.684920
8	7	0	-3.646248	-0.303734	-1.236558
9	7	0	-3.092219	-1.893194	0.384371
10	6	0	-3.816969	-1.468042	-0.636699
11	1	0	-4.625947	-2.122265	-1.002384
12	29	0	-1.520230	-0.928824	0.899167
13	7	0	-0.578469	-0.305691	2.442670
14	7	0	1.697274	-0.848898	1.990082
15	6	0	0.700100	-0.389412	2.739822
16	1	0	0.983693	-0.039649	3.748115
17	29	0	1.343790	1.230237	-0.578684

18	7	0	0.499051	2.678160	0.418184
19	7	0	-1.851977	2.560426	0.016066
20	6	0	-0.746327	3.107244	0.501828
21	1	0	-0.890077	4.057176	1.050795
22	6	0	-4.473869	-0.031939	-2.396351
23	1	0	-4.999363	0.928518	-2.289539
24	1	0	-3.869262	0.030583	-3.312787
25	1	0	-5.236072	-0.813066	-2.558509
26	6	0	-3.439345	-3.183641	0.949422
27	1	0	-4.277501	-3.662441	0.414883
28	1	0	-2.586571	-3.875410	0.910249
29	1	0	-3.729664	-3.091175	2.007286
30	6	0	-1.410219	0.387947	3.416305
31	1	0	-1.815746	1.314349	2.987642
32	1	0	-2.264556	-0.235999	3.711320
33	1	0	-0.852185	0.657666	4.329817
34	6	0	2.979148	-0.959884	2.677622
35	1	0	3.774086	-0.514737	2.072536
36	1	0	2.966568	-0.459898	3.659548
37	1	0	3.250770	-2.013242	2.852082
38	6	0	4.622023	-2.036620	-0.399179
39	1	0	5.582936	-1.745000	-0.855810
40	1	0	4.836779	-2.414385	0.612290
41	1	0	4.208802	-2.871512	-0.980226
42	6	0	3.769986	2.371364	-1.813321
43	1	0	3.809220	3.253985	-1.155627
44	1	0	4.796129	2.183538	-2.173648
45	1	0	3.161877	2.652270	-2.684381
46	6	0	1.458097	3.430477	1.216926
47	1	0	1.864598	2.812504	2.030752
48	1	0	2.310830	3.755336	0.606654
49	1	0	1.012681	4.330016	1.678039
50	6	0	-3.049566	3.383787	0.147428
51	1	0	-2.867218	4.301559	0.734785
52	1	0	-3.429625	3.697010	-0.836535
53	1	0	-3.857011	2.826872	0.639455
54	7	0	0.665077	-2.478618	-2.249101
55	7	0	-0.213487	-1.603639	-2.262770

56	8	0	1.581950	-2.549159	-1.294610

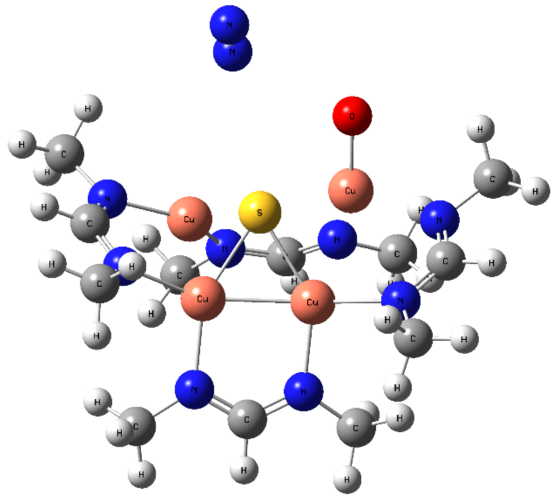

$[Cu_4S(NCN)_4O]^-$ + N_2 (Sum of electronic and thermal Free Energies= -8056.115109 Hartrees)

Center Number	Atomic Number	Atomic Type	Coordinates (Angstroms) X	Y	Z
1	29	0	-1.866880	1.326090	-0.154469
2	16	0	0.159632	0.505993	-1.184041
3	7	0	-3.659293	0.428143	-0.509013
4	7	0	-2.671468	-1.597804	-1.105371
5	6	0	-3.680063	-0.741693	-1.102629
6	1	0	-4.604284	-1.039234	-1.634830
7	29	0	1.434563	-1.108839	-0.494053
8	7	0	3.144828	-0.637913	-1.333167
9	7	0	3.259772	1.376033	-0.144295
10	6	0	3.735330	0.498108	-1.007632
11	1	0	4.704864	0.720829	-1.484218
12	29	0	1.505811	1.143135	0.603113
13	7	0	0.658252	1.130699	2.318642
14	7	0	-1.668005	1.015811	1.799898
15	6	0	-0.625570	1.008857	2.613969
16	1	0	-0.856636	0.883906	3.687156
17	29	0	-0.878925	-1.242516	-0.361607

18	7	0	-0.639228	-2.799877	0.773375
19	7	0	1.672571	-2.743424	0.531657
20	6	0	0.568519	-3.266609	1.030760
21	1	0	0.658231	-4.140788	1.699277
22	6	0	3.733042	-1.430335	-2.391182
23	1	0	3.851173	-2.477759	-2.078446
24	1	0	3.098499	-1.432336	-3.290761
25	1	0	4.727871	-1.058090	-2.688589
26	6	0	4.075449	2.545332	0.126810
27	1	0	4.998144	2.563562	-0.477880
28	1	0	3.521326	3.469085	-0.088459
29	1	0	4.371234	2.588801	1.186021
30	6	0	1.563586	1.033671	3.456440
31	1	0	2.226258	0.161574	3.364102
32	1	0	2.206950	1.922090	3.523161
33	1	0	1.025502	0.938939	4.416556
34	6	0	-2.962143	0.902958	2.465371
35	1	0	-3.565987	0.125246	1.988368
36	1	0	-2.860618	0.662936	3.536178
37	1	0	-3.530193	1.843039	2.390403
38	6	0	-4.836787	1.263390	-0.626866
39	1	0	-5.673937	0.741212	-1.122471
40	1	0	-5.189074	1.585196	0.365503
41	1	0	-4.604575	2.169971	-1.198951
42	6	0	-2.809664	-2.784850	-1.923809
43	1	0	-2.442717	-3.671249	-1.388235
44	1	0	-3.858754	-2.974480	-2.208406
45	1	0	-2.226491	-2.713430	-2.856151
46	6	0	-1.746944	-3.369451	1.518926
47	1	0	-2.027430	-2.738334	2.376575
48	1	0	-2.631278	-3.444338	0.878209
49	1	0	-1.512626	-4.374597	1.907276
50	6	0	2.929206	-3.398087	0.829744
51	1	0	2.822253	-4.171736	1.608555
52	1	0	3.357228	-3.886489	-0.060058
53	1	0	3.670179	-2.668935	1.180946
54	7	0	0.449869	4.348448	-0.726679
55	7	0	0.563679	4.521653	-1.812363

56	8	0	-1.911549	2.505144	-1.408863

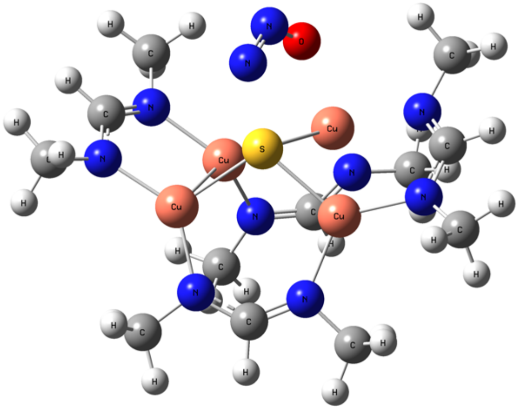

$[Cu_4S(NCN)_4{\cdot}N_2O]^{2-}$ (Sum of electronic and thermal Free Energies= -8056.023196 Hartrees)

Center Number	Atomic Number	Atomic Type	Coordinates (Angstroms) X	Y	Z
1	29	0	1.322767	-1.153420	0.155424
2	16	0	-0.156893	-0.134094	-1.277041
3	7	0	3.334411	-1.121662	-0.519332
4	7	0	3.319902	1.150009	-1.069111
5	6	0	3.886636	-0.053383	-1.036662
6	1	0	4.897792	-0.148126	-1.493189
7	29	0	-2.114703	0.841058	-0.641773
8	7	0	-3.678631	-0.289004	-0.915928
9	7	0	-2.638024	-1.981529	0.313289
10	6	0	-3.588752	-1.540140	-0.477937
11	1	0	-4.371431	-2.255011	-0.802749
12	29	0	-1.112327	-0.828649	0.787722
13	7	0	-0.560596	-0.204115	2.534246
14	7	0	1.613863	-0.931015	2.106210
15	6	0	0.698847	-0.387947	2.886468
16	1	0	1.004331	-0.056420	3.899723
17	29	0	1.430966	1.266626	-0.571586
18	7	0	0.574186	2.875000	0.170149

19	7	0	-1.802888	2.639910	-0.006855
20	6	0	-0.679436	3.263758	0.322006
21	1	0	-0.815721	4.259458	0.794515
22	6	0	-4.805966	0.024103	-1.758878
23	1	0	-5.421988	0.838638	-1.336629
24	1	0	-4.484912	0.363515	-2.757650
25	1	0	-5.478055	-0.843231	-1.913859
26	6	0	-2.606202	-3.398649	0.598092
27	1	0	-3.445642	-3.947892	0.127513
28	1	0	-1.666819	-3.836687	0.235574
29	1	0	-2.653063	-3.585632	1.684177
30	6	0	-1.398505	0.552047	3.432073
31	1	0	-1.674105	1.527451	3.000307
32	1	0	-2.341986	0.020037	3.635213
33	1	0	-0.911437	0.750067	4.407949
34	6	0	2.910334	-1.158345	2.703230
35	1	0	3.701096	-0.830166	2.017966
36	1	0	3.041741	-0.622894	3.664974
37	1	0	3.089624	-2.230913	2.906370
38	6	0	4.046944	-2.367926	-0.665219
39	1	0	4.991578	-2.267982	-1.239309
40	1	0	4.306805	-2.797900	0.318512
41	1	0	3.412919	-3.108313	-1.170685
42	6	0	4.120090	2.219125	-1.612284
43	1	0	4.399504	2.969253	-0.848238
44	1	0	5.066255	1.858641	-2.064366
45	1	0	3.577717	2.769251	-2.397971
46	6	0	1.558882	3.803002	0.701861
47	1	0	2.167910	3.328771	1.485160
48	1	0	2.258419	4.136434	-0.079322
49	1	0	1.100179	4.710345	1.144594
50	6	0	-3.015786	3.388740	0.274754
51	1	0	-2.814516	4.373102	0.745516
52	1	0	-3.593696	3.581040	-0.643518
53	1	0	-3.679248	2.829739	0.950586
54	7	0	0.133604	-2.766047	-1.934426
55	7	0	-0.345226	-1.719586	-2.386161
56	8	0	0.769492	-2.863990	-0.781492

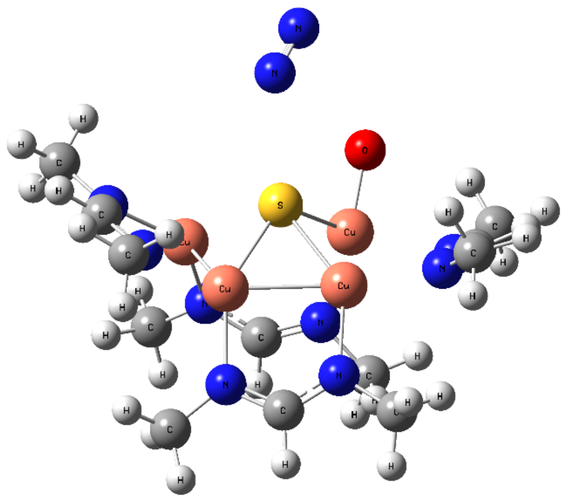

$[Cu_4S(NCN)_4{\cdot}O]^{2-} + N_2$ (Sum of electronic and thermal Free Energies= -8056.041476 Hartrees)

Center Number	Atomic Number	Atomic Type	Coordinates (Angstroms) X	Y	Z
1	29	0	-1.504517	1.340190	0.188459
2	16	0	0.079577	0.551729	-1.162060
3	7	0	-3.573409	0.766194	0.228671
4	7	0	-3.100901	-1.146100	-1.047440
5	6	0	-3.873125	-0.127497	-0.684204
6	1	0	-4.852566	-0.028289	-1.206320
7	29	0	1.246061	-1.152783	-0.379468
8	7	0	3.010522	-1.163529	-1.353607
9	7	0	3.510282	1.008666	-0.642985
10	6	0	3.764587	-0.083106	-1.346917
11	1	0	4.686554	-0.088381	-1.965004
12	29	0	1.848248	1.098984	0.347019
13	7	0	1.259378	1.315599	2.160675
14	7	0	-1.029803	0.662984	2.023352
15	6	0	0.102866	0.893015	2.657556
16	1	0	0.090927	0.712887	3.753342
17	29	0	-1.129371	-1.252801	-0.623302

18	7	0	-0.989698	-3.029869	0.239576
19	7	0	1.224744	-2.664946	0.870419
20	6	0	0.096483	-3.341380	0.929578
21	1	0	0.051321	-4.221436	1.602897
22	6	0	3.443069	-2.265871	-2.174751
23	1	0	3.570439	-3.184629	-1.578338
24	1	0	2.698892	-2.502397	-2.952408
25	1	0	4.405874	-2.070385	-2.688751
26	6	0	4.425174	2.114528	-0.805874
27	1	0	5.273233	1.871675	-1.476865
28	1	0	3.919540	2.996959	-1.228591
29	1	0	4.851563	2.430577	0.160822
30	6	0	2.241231	1.705260	3.155201
31	1	0	3.206762	1.213457	2.972082
32	1	0	2.430925	2.792146	3.137706
33	1	0	1.926414	1.450893	4.188425
34	6	0	-2.031894	-0.051364	2.797461
35	1	0	-2.054628	-1.117568	2.524330
36	1	0	-1.837469	0.009648	3.885231
37	1	0	-3.025778	0.351410	2.586314
38	6	0	-4.502786	1.858290	0.393629
39	1	0	-5.447782	1.711494	-0.167483
40	1	0	-4.770772	1.996732	1.456837
41	1	0	-4.019319	2.778892	0.035509
42	6	0	-3.583323	-1.932747	-2.158192
43	1	0	-3.508670	-3.012731	-1.950414
44	1	0	-4.643139	-1.720621	-2.401731
45	1	0	-3.003087	-1.752012	-3.080001
46	6	0	-2.116303	-3.925759	0.390679
47	1	0	-3.044398	-3.345115	0.449392
48	1	0	-2.222305	-4.616849	-0.466684
49	1	0	-2.047687	-4.554248	1.299863
50	6	0	2.299861	-3.071175	1.740504
51	1	0	2.107458	-4.040442	2.241627
52	1	0	3.240028	-3.169552	1.176867
53	1	0	2.485877	-2.325556	2.531182
54	7	0	-0.059986	4.495306	-1.271857
55	7	0	-0.564977	5.386399	-1.704436

56	8	0	-1.811470	2.697947	-0.822496

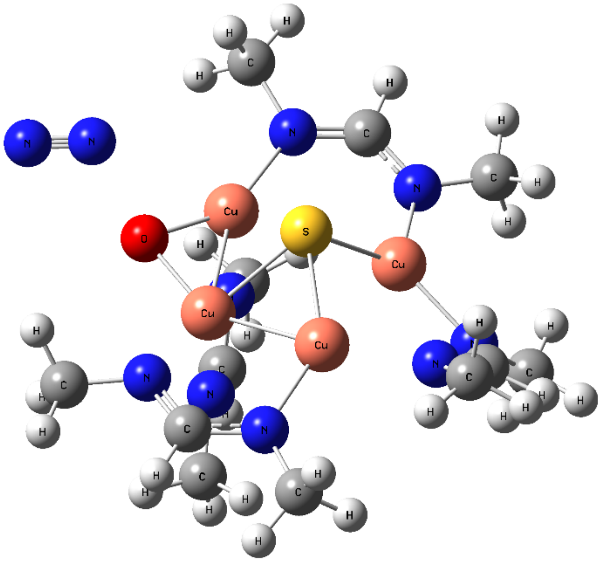

$[Cu_4S(NCN)_4(\mu\text{-}O)]^{2-} + N_2$ (Sum of electronic and thermal Free Energies= -8056.063793 Hartrees)

Center Number	Atomic Number	Atomic Type	Coordinates (Angstroms) X	Y	Z
1	29	0	1.494333	-1.474961	-0.129385
2	16	0	0.144482	0.061156	-1.364978
3	7	0	3.310142	-0.924946	-0.658720
4	7	0	3.230421	1.405356	-0.277368
5	6	0	3.832210	0.283008	-0.642156
6	1	0	4.889787	0.369274	-0.973486
7	29	0	-1.726052	0.954971	-0.605614
8	7	0	-3.498452	0.492734	0.069930
9	7	0	-2.947542	-1.772287	0.148362
10	6	0	-3.801754	-0.794228	0.265412
11	1	0	-4.847724	-1.031265	0.558270
12	29	0	-0.936072	-1.210276	0.118057
13	7	0	-0.627287	-0.941671	2.191970
14	7	0	1.690451	-1.233489	1.954290
15	6	0	0.602344	-0.992396	2.660886

16	1	0	0.738965	-0.805832	3.752454
17	29	0	1.282483	1.601855	-0.205348
18	7	0	0.419944	3.271050	0.322435
19	7	0	-1.632555	2.961851	-0.826321
20	6	0	-0.710774	3.692865	-0.223364
21	1	0	-0.911420	4.787293	-0.161228
22	6	0	-4.584148	1.427011	0.265641
23	1	0	-4.225452	2.331636	0.775432
24	1	0	-5.046817	1.764940	-0.682428
25	1	0	-5.403339	1.006695	0.880186
26	6	0	-3.389437	-3.110873	0.441693
27	1	0	-4.479482	-3.174807	0.640090
28	1	0	-3.156489	-3.787010	-0.392997
29	1	0	-2.861258	-3.513864	1.319231
30	6	0	-1.675981	-0.667490	3.137986
31	1	0	-2.254268	0.221954	2.843125
32	1	0	-2.400783	-1.499497	3.196748
33	1	0	-1.298766	-0.493352	4.167506
34	6	0	2.945317	-1.196067	2.653922
35	1	0	3.618635	-0.433144	2.232483
36	1	0	2.832638	-0.972359	3.735602
37	1	0	3.482856	-2.160469	2.582939
38	6	0	4.130464	-1.977239	-1.218233
39	1	0	5.156078	-1.634120	-1.459937
40	1	0	4.219325	-2.822985	-0.519707
41	1	0	3.688803	-2.379430	-2.143248
42	6	0	4.083734	2.572470	-0.242850
43	1	0	4.235231	2.944934	0.786169
44	1	0	5.091909	2.384943	-0.667113
45	1	0	3.641106	3.402638	-0.813342
46	6	0	1.071887	4.231156	1.190156
47	1	0	0.984310	3.943455	2.251714
48	1	0	2.145613	4.298613	0.971848
49	1	0	0.645760	5.252016	1.098229
50	6	0	-2.590458	3.710008	-1.608316
51	1	0	-2.633631	4.782438	-1.325785
52	1	0	-2.359053	3.677067	-2.688475
53	1	0	-3.601160	3.298338	-1.492708

54	7	0	-0.282202	-3.853145	-2.390377
55	7	0	-0.565774	-4.909578	-2.193544
56	8	0	0.163804	-2.694146	-0.156844

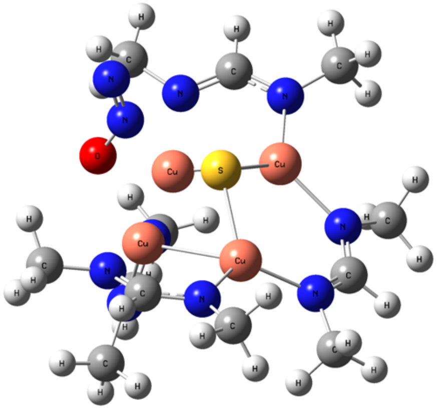

$[Cu_4S(NCN)_4{\cdot}N_2O]^-$ isomer (Sum of electronic and thermal Free Energies= -8056.004321 Hartrees)

Center Number	Atomic Number	Atomic Type	Coordinates (Angstroms) X	Y	Z
1	29	0	1.286556	-1.082346	0.236256
2	16	0	0.000379	0.148080	-1.346530
3	7	0	3.177252	-1.496237	-0.375422
4	7	0	3.632380	0.764965	-0.747404
5	6	0	3.975584	-0.519854	-0.734678
6	1	0	5.009063	-0.776153	-1.053079
7	29	0	-1.728009	1.194206	-0.461643
8	7	0	-3.631220	0.768041	-0.747206
9	7	0	-3.178740	-1.493926	-0.376673
10	6	0	-3.975920	-0.516377	-0.735361
11	1	0	-5.009649	-0.771298	-1.054032
12	29	0	-1.288153	-1.082632	0.236154
13	7	0	-1.167898	-1.243204	2.208156
14	7	0	1.166407	-1.242907	2.208327
15	6	0	-0.000777	-1.266175	2.822048
16	1	0	-0.000860	-1.305439	3.932253

17	29	0	1.729678	1.193541	-0.462058
18	7	0	1.196950	2.914646	0.347050
19	7	0	-1.194107	2.915042	0.347646
20	6	0	0.001571	3.409585	0.622518
21	1	0	0.001875	4.379902	1.164950
22	6	0	-4.656745	1.699630	-1.149562
23	1	0	-4.918720	2.406647	-0.342004
24	1	0	-4.332531	2.316684	-2.004099
25	1	0	-5.597070	1.197454	-1.453494
26	6	0	-3.692320	-2.838079	-0.471315
27	1	0	-4.719710	-2.882731	-0.887605
28	1	0	-3.044916	-3.457335	-1.109102
29	1	0	-3.715981	-3.328691	0.516740
30	6	0	-2.343720	-1.261415	3.044463
31	1	0	-2.972689	-0.374670	2.870299
32	1	0	-2.979403	-2.137406	2.830389
33	1	0	-2.103052	-1.288282	4.126706
34	6	0	2.342146	-1.261054	3.044738
35	1	0	2.971006	-0.374201	2.870738
36	1	0	2.101375	-1.288093	4.126953
37	1	0	2.977967	-2.136921	2.830593
38	6	0	3.689374	-2.841026	-0.468758
39	1	0	4.716547	-2.887268	-0.885414
40	1	0	3.712917	-3.330596	0.519830
41	1	0	3.041013	-3.460261	-1.105580
42	6	0	4.658895	1.695064	-1.150677
43	1	0	4.921217	2.402889	-0.343951
44	1	0	5.598864	1.191613	-1.453607
45	1	0	4.335579	2.311306	-2.006153
46	6	0	2.308782	3.731143	0.797125
47	1	0	2.978513	3.160074	1.456780
48	1	0	2.925663	4.077692	-0.047859
49	1	0	1.984428	4.633019	1.355035
50	6	0	-2.305420	3.731904	0.798340
51	1	0	-1.980469	4.633499	1.356353
52	1	0	-2.922478	4.078926	-0.046318
53	1	0	-2.975134	3.160950	1.458106
54	7	0	-0.001022	-2.171519	-2.008362

55	7	0	-0.001535	-2.651019	-3.067289
56	8	0	-0.001187	-2.626015	-0.753321

Experimental Details for X-ray Spectroscopy

All data were measured at the Stanford Synchrotron Radiation Lightsource (SSRL) under ring conditions of 3.0 GeV and 500 mA. Samples were prepared in an inert-atmosphere glovebox and were measured as solids. For Cu K-edge measurements, samples were ground with BN to a final concentration of 5 weight % Cu, pressed into 1 mm aluminum spacers and sealed with 37μm Kapton tape. For S K-edge measurements, samples were prepared by grinding to a fine powder and spreading thinly onto 38 μm low-S Mylar tape.

Cu K-edge measurements were carried out at either SSRL Beamline 9-3. Beamline 9-3 is equipped with a 16-pole, 2-Tesla wiggler source. Incident X-ray radiation was monochromated using a double Si(220) crystal monochromator. Samples were maintained at 10 K in a liquid He cryostat during data collection. Spectra were collected in fluorescence mode, with X-rays detected by a passivated implanted planar silicon (PIPS) detector placed at a 90° angle to the sample. Inelastic scatter was attenuated using a Soller slits fitted with a Ni filter. A Cu foil and a third ionization chamber upstream of the sample were used for internal energy calibration, setting the first inflection point of the Cu foil scan to 8980.3 eV. Data were collected from 8660 eV to 9380 eV. Three scans were measured and averaged for each compound. Spectra were processed using Sixpack[3] and Igor Pro. The region below 8970 eV was used to fit a linear background, while the region above 9000 eV was flattened with a piecewise spline and set to an average intensity of 1.0.

S K-edge measurements were carried out at SSRL Beamline 4-3, which is equipped with a 20-pole, 2 Tesla wiggler source. All samples were measured in a He atmosphere at room temperature in fluorescence mode using a Lytle detector. Intensity was normalized with respect to the incident beam using a He-filled ion chamber upstream of the sample. The incident beam energy was calibrated by setting the first inflection point in the S K-edge spectrum of $Na_2S_2O_3$ to 2472.02 eV. Data were collected from 2420 to 2700 eV. Three scans were measured and averaged for each compound. The region below 2460 eV was used to fit a linear background, and the region above 2475 eV was flattened with a piecewise spline and set to an average intensity of 1. Raw data was processed using Sixpack and Igor Pro. An in-house developed, Monte-Carlo based, nonlinear least squares fitting algorithm was used to fit the S K-edge XAS spectra in Python as described previously for N K-edge XAS.[4]

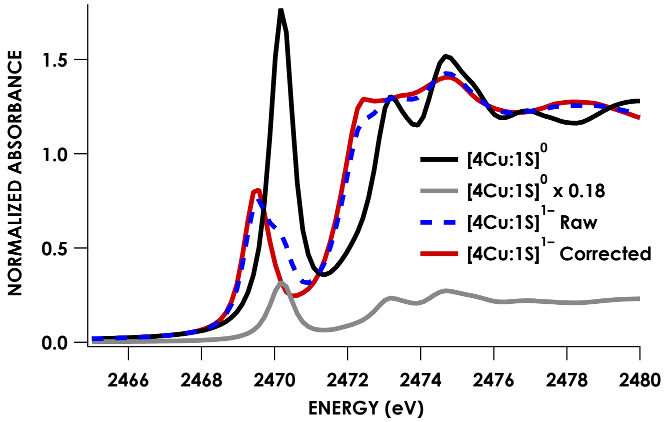

Figure S16. The raw S K-edge XAS obtained for the $[4Cu{:}1S]^{1-}$ cluster contained a ca. 18% impurity of $[4Cu{:}1S]^{0}$. The proportion of impurity was determined by iteratively subtracting portions of the $[4Cu{:}1S]^{0}$ spectrum until the second derivative of the corrected $[4Cu{:}1S]^{1-}$ spectrum gave a single minimum in the pre-edge region.

Computational Details for X-ray Spectroscopy

Calculations carried out to facilitate XAS interpretation were carried out using version 4.0 of the ORCA quantum chemistry package.[5] For computational expediency, electronic structure calculations were carried out on crystallographic structures with NCN ligands whose mesityl substituents were truncated to methyl groups. H-atom positions of these structures were optimized using the BP86 generalized gradient approximation (GGA) density functional[6] with the scalar relativistically recontracted ZORA-def2-TZVP(-f)[7] basis set on all atoms. Solvation was modeled with the conductor-like polarizable continuum model (CPCM)[57] in the dielectric of CH_2Cl_2 (9.08). The zeroth-order regular approximation (ZORA)[9] as implemented by van Wüllen[10] was used to model relativistic effects.

TDDFT calculations[11] of the S K-edge XAS of the $[4Cu{:}1S]^{1-/0}$ redox series were carried out using the B3LYP hybrid density functional[12] with the CP(PPP) basis set[13] on Cu with a special grid accuracy of ORCA Grid7 and ZORA-def2-TZVP(-f)[7] basis set with a grid accuracy of ORCA Grid7 on S and ORCA Grid4 on all other atoms. The RIJCOSX approximation[14] was used to speed the calculation of Hartree-Fock exchange. A total of 100 roots were calculated with the S 1s orbital serving as the sole donor orbital and all vacancies allowed as acceptors. A 1 eV Gaussian line broadening was applied to all transitions to produce the final, plotted spectra. Calculated energies were shifted by +40.4 eV to align calculated data with experiment.

H-Atom Optimized Truncated Model of $[4Cu:1S]^0$

Cu	-0.16712034498453	8.96786482433254	6.11520027228238
S	0.00094742532157	10.91177901281958	7.08515097396027
N	1.32125950507055	7.97309368900697	6.91516644755009
N	2.91450882321073	9.55216114905658	6.16060399143354
C	2.56366591700838	8.41714564585269	6.76364707087451
H	3.37628897367932	7.78917993059245	7.16946199906332
C	4.29975709876559	9.85662810328748	6.04820940596390
Cu	-1.69883048725913	10.77703976118424	5.70336987733961
N	-2.55368908864038	9.63250843515467	4.37313165049402
N	-1.22688853217347	7.81164068122838	5.01638388328573
C	-2.21172564118221	8.34840873538204	4.30182861125846
H	-2.77287520048941	7.69328325946795	3.61223393418429
C	-1.04863231047925	6.40100626037658	4.99940270304433
Cu	0.16853485025717	12.85606293978586	6.11523169624865
N	-1.32056326591928	13.85003560101613	6.91493558823146
N	-2.91452485491438	12.26906641356324	6.16542810430978
C	-1.03018386207460	15.08700555130366	7.54139383892603
C	-2.56292488315693	13.40480760172095	6.76657497050799
H	-3.37497707885279	14.03212255654098	7.17445857610008
C	-4.29975213975465	11.96233501569793	6.05860274441652
Cu	1.69920050388744	11.04540961135531	5.70182217262509
N	2.55265968153641	12.18910456591208	4.36979047557958
N	1.22781070078539	14.01088152105834	5.01520813678770
C	2.21114815067777	13.47324205589540	4.29922352587709
H	2.77199797811610	14.12819987563306	3.60918308852714
C	1.05298204043031	15.42196044722632	5.00072916849447
C	1.03010934056401	6.73730815623383	7.54343821189094
H	0.12557235948532	6.82026381710840	8.16615017745468
H	1.84893459935065	6.40497185355537	8.20923651212140
H	0.85270189074219	5.92174550892748	6.82269812686469
H	-1.67334900045114	5.91923115009483	4.22418695480199
H	-1.32083886386218	5.93909310901822	5.96345128650384
H	-0.00184760590852	6.12908330392414	4.79041410782954
C	3.58375792872827	11.70608421179314	3.52448207469924
H	3.69866218673609	12.32648623685364	2.61647480018969
H	3.36706494294488	10.68172842601468	3.18686366416063

H	4.56862780896281	11.67956432654975	4.02166994086873
H	4.49618273708894	10.91166026707746	6.29380041928239
H	4.68372793275555	9.68549918614894	5.02832341857510
H	4.91941450210187	9.24601382966963	6.73130632662218
H	1.67988350012992	15.90371413715653	4.22726194216942
H	1.32533278726925	15.88127326312442	5.96595910064939
H	0.00716419100349	15.69704880623189	4.79100624331404
H	-0.12583799472373	15.00559493675608	8.16463174914842
H	-1.84956077208833	15.42024903750503	8.20601888852638
H	-0.85280779567864	15.90131520660660	6.81925527329181
C	-3.58763223689624	10.11514012570801	3.53137029146966
H	-3.70302878722921	9.49683603361224	2.62199304506825
H	-3.37400183831410	11.14087492860645	3.19605446609778
H	-4.57164069057182	10.13828215204932	4.03053172119644
H	-4.49346184310415	10.90699702778337	6.30503048167494
H	-4.68802178923379	12.13288609701682	5.04024781751798
H	-4.91773144866709	12.57201162042223	6.74410005064440

H-Atom Optimized Truncated Model of $[4Cu:1S]^{1-}$

Cu	7.03453113740897	7.55993973700832	11.05209362594193
Cu	9.47559039409535	7.22869884113902	10.88045109096620
Cu	8.96917609016007	7.61962029144022	8.24186262751464
Cu	6.51307031376902	7.37397611633347	8.39096489829336
S	7.97220485510672	6.18550118463537	9.59780835279663
N	7.38364684724101	8.91172040119965	12.41347861341600
N	9.64450372531640	8.30749224073309	12.49247419691284
N	11.24968319963926	6.52073406168340	10.29413006572949
N	10.84013556936995	6.92359771581322	8.00580364977950
N	8.67534108355277	9.06356870507787	6.96094989453601
N	6.39283392526912	8.54796572110296	6.84439374640637
N	4.71385084782665	6.70115572364220	8.94272739858532
N	5.13523949826517	6.93648829186997	11.25181244312673
C	8.59903121296441	8.99605315762092	12.94425529512397
H	8.75209986329926	9.67953115688900	13.80244171629646
C	6.35025288178661	9.76254832842535	12.86806525025677
C	10.86987522949424	8.38475797200440	13.20209296797282
C	11.57357070757024	6.44598293760471	9.00630418164364

H	12.52133850135693	5.92809748206697	8.74707038631889
C	11.29291487692464	6.71605329507202	6.68119145220354
C	7.46565763830179	9.21954307125744	6.43317270851326
H	7.34286731598966	9.95182532697273	5.61125458303900
C	9.73711861056280	9.89894476737125	6.54305172438304
C	5.18016472133609	8.70452901465385	6.12596136285546
C	4.38387941753758	6.55686843683470	10.22284891616851
H	3.41690188268257	6.05903640412195	10.44832021327123
C	3.83714991876594	6.24459034439722	7.93230333814167
C	4.67367304955783	6.67017601435347	12.56252696014419
H	9.42518559247431	10.62461482556143	5.76508469542065
H	10.58079443539921	9.32406126783157	6.12712332514499
H	10.14867526016778	10.48931114276728	7.38215446499560
H	10.52300135984795	6.22539468554427	6.05797893280268
H	12.19674773899361	6.07436417672314	6.63249857889314
H	11.54989101955935	7.65968808839939	6.16523476719763
C	12.11250170442186	5.97347878835987	11.26998944787322
H	12.90046571920146	5.32817203556993	10.82814549502925
H	11.55884865374053	5.34799076408306	11.99255666660225
H	12.63368554393406	6.74439960330587	11.86676363304763
H	11.70786309550291	8.64007102628213	12.53182552818208
H	11.13649948695993	7.42511029042553	13.68167824110703
H	10.84568040131011	9.14895182393129	14.00443668422985
H	5.48300579301647	9.19911756379213	13.24894227691069
H	5.96824370267652	10.41076560338081	12.05821511018203
H	6.68191644092665	10.43538319491228	13.68479983029382
H	3.75509124648444	6.04801382161376	12.57500666231676
H	4.43703148408244	7.59144573740967	13.12691651500946
H	5.43158110578411	6.13098177730917	13.15907842575013
H	4.37358049556054	5.64512059438439	7.17578316838019
H	3.34809995831152	7.06962208401423	7.38308957926065
H	3.02406207999242	5.60275150743267	8.33147194750251
H	5.23944598582331	9.51131547825375	5.36806431375095
H	4.34232569874404	8.95311470154421	6.79873779476944
H	4.88808268193335	7.78341267584340	5.58911225500993

References

[1] (a) Johnson, B. J.; Antholine, W. E.; Lindeman, S. V; Mankad, N. P. *Chem. Commun.* **2015**, *51*, 11860. (b) Johnson, B. J.; Antholine, W. E.; Lindeman, S. V; Graham, M. J.; Mankad, N. P. *J. Am. Chem. Soc.* **2016**, *138*, 13107.

[2] Gaussian 16, Revision B.01, Frisch, M. J.; Trucks, G. W.; Schlegel, H. B.; Scuseria, G. E.; Robb, M. A.; Cheeseman, J. R.; Scalmani, G.; Barone, V.; Petersson, G. A.; Nakatsuji, H.; Li, X.; Caricato, M.; Marenich, A. V.; Bloino, J.; Janesko, B. G.; Gomperts, R.; Mennucci, B.; Hratchian, H. P.; Ortiz, J. V.; Izmaylov, A. F.; Sonnenberg, J. L.; Williams-Young, D.; Ding, F.; Lipparini, F.; Egidi, F.; Goings, J.; Peng, B.; Petrone, A.; Henderson, T.; Ranasinghe, D.; Zakrzewski, V. G.; Gao, J.; Rega, N.; Zheng, G.; Liang, W.; Hada, M.; Ehara, M.; Toyota, K.; Fukuda, R.; Hasegawa, J.; Ishida, M.; Nakajima, T.; Honda, Y.; Kitao, O.; Nakai, H.; Vreven, T.; Throssell, K.; Montgomery, J. A., Jr.; Peralta, J. E.; Ogliaro, F.; Bearpark, M. J.; Heyd, J. J.; Brothers, E. N.; Kudin, K. N.; Staroverov, V. N.; Keith, T. A.; Kobayashi, R.; Normand, J.; Raghavachari, K.; Rendell, A. P.; Burant, J. C.; Iyengar, S. S.; Tomasi, J.; Cossi, M.; Millam, J. M.; Klene, M.; Adamo, C.; Cammi, R.; Ochterski, J. W.; Martin, R. L.; Morokuma, K.; Farkas, O.; Foresman, J. B.; Fox, D. J. Gaussian, Inc., Wallingford CT, 2016.

[3] Webb, S. M. *Phys. Scr.* **2005**, 1011.

[4] (a) Walroth, R. C.; Miles, K. C.; Lukens, J. T.; MacMillan, S. N.; Stahl, S. S.; Lancaster, K. M. *J. Am. Chem. Soc.* **2017**, *139*, 13507–13517. (b) Lukens, J. T.; DiMucci, I. M.; Kurogi, T.; Mindiola, D. J.; Lancaster, K. M. *Chem. Sci.* **2019,** *10*, 5044-5055. (c) Delgado-Jaime, M. U.; Mewis, C. P.; Kennepohl, P. *J. Synch. Rad.* **2010**, *17*, 132-137. (d) Hunter, J. D., *Comput. Sci. Eng.* **2007**, *9*, 90-95. € McKinney, W., *Proc. of the 9th Python in Science Conf.* **2010**, 51 - 56. (f) Walt, S. v. d.; Colbert, S. C.; Varoquaux, G., *Comput. Sci. Eng.* **2011**, *13*, 22-30. (g) Jones, E.; Oliphant, T.; Peterson, P. SciPy: Open Source Scientific Tools for Python. (f) Oliphant, T. E., *Comput. Sci. Eng.* **2007**, *9*, 10-20. (g) Millman, K. J.; Aivazis, M., *Comput. Sci. Eng.* **2011**, 13, 9-12.

[5] Neese, F. *WIREs Comput. Mol. Sci.* **2012,** *2*, 73-78.

[6] (a) Becke, A. D. *Phys. Rev. A: At., Mol., Opt. Phys.* **1988**, *38*, 3098−3100. (b) Perdew, J. P. *Phys. Rev. B: Condens. Matter Mater. Phys.* **1986**, *33*, 8822−8824.

[7] Weigend, F.; Ahlrichs, R. *Phys. Chem. Chem. Phys.* **2005,** *7*, 3297-3305.

[8] Takano, Y.; Houk, K. N. *J. Chem. Theor. Comput.* **2005**, *1*, 70-77.

[9] van Lenthe, E.; van der Avoird, A.; Wormer, P. E. S. *J. Chem. Phys.* **1998,** *108*, 4783-4796.

[10] van Wüllen, C. *J. Chem. Phys.* **1998**, *109*, 392-399.

[11] (a) Gross, E. K. U.; Kohn, W. *Adv. Quantum Chem.* **1990**, 255-291. (b) Debeer George, S.; Neese, F. Calibration of scalar relativistic density functional theory for the calculation of sulfur K-edge X-ray absorption spectra. *Inorg. Chem.* **2010,** *49*, 1849-1853.

[12] Stephens, P.; Devlin, F.; Chabalowski, C.; Frisch, M. J. *J. Phys. Chem.* **1994**, *98*, 11623−11627.

[13] Neese, F. *Inorg. Chim. Acta.* **2002,** *337*, 181-192.

[14] Neese, F.; Wennmohs, F.; Hansen, A.; Becker, U. *Chem. Phys.* **2009,** *356*, 98-109.
