# Supplementary material for: Probing the electronic and mechanistic roles of the μ4-sulfur atom in a synthetic CuZ model system
Source: Chem Sci. 2020 Feb 17;11(13):3441–7. doi: 10.1039/c9sc06251c (PMC8515425; doi:10.1039/c9sc06251c)

Electronic Supplementary Material (ESI) for Chemical Science.
This journal is © The Royal Society of Chemistry 2020

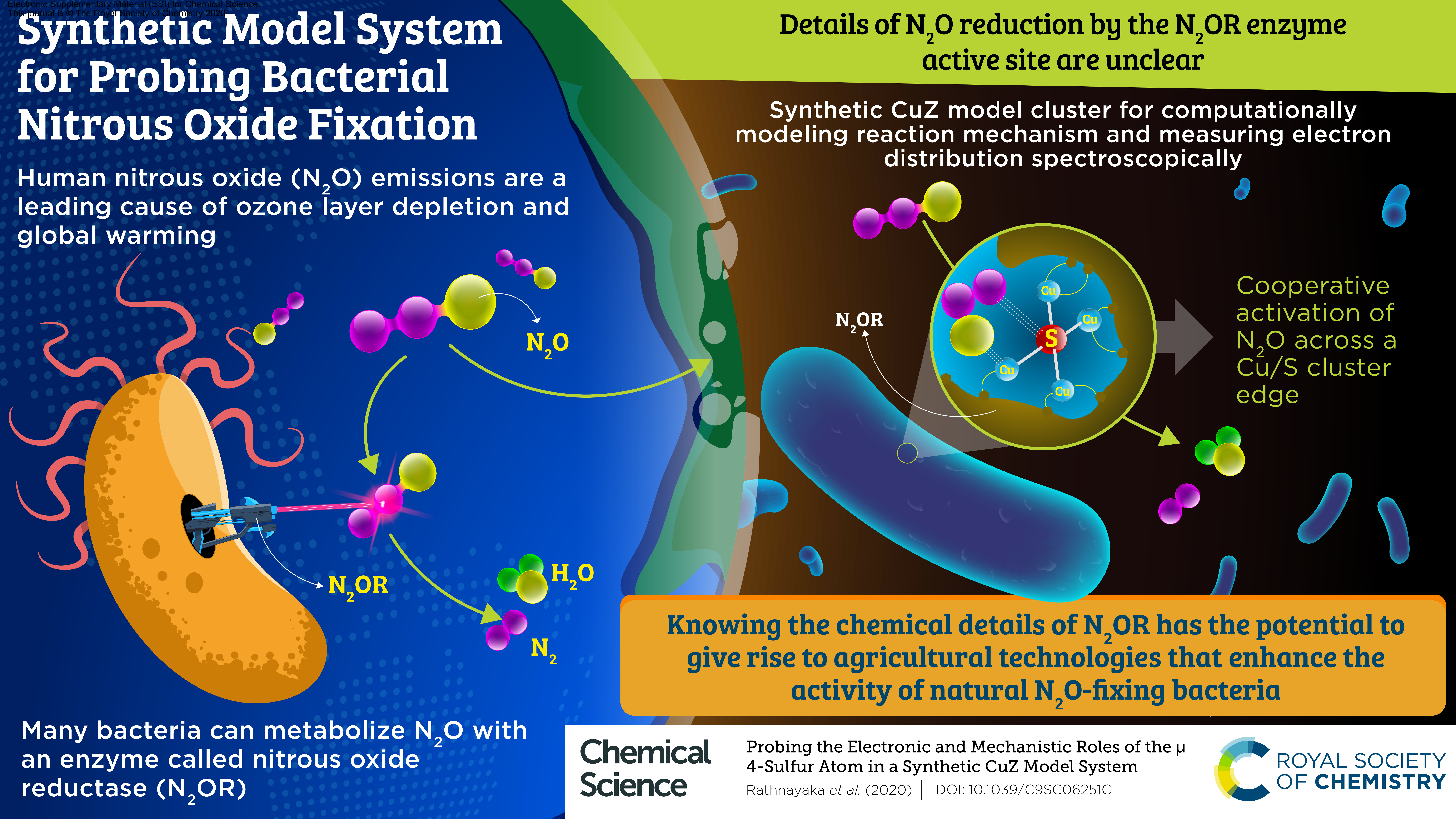

Supplement: SC-011-C9SC06251C-s002 [file SC-011-C9SC06251C-s002.pdf]
